# Effects of a Plant Sterol or Stanol Enriched Mixed Meal on Postprandial Lipid Metabolism in Healthy Subjects

**DOI:** 10.1371/journal.pone.0160396

**Published:** 2016-09-09

**Authors:** Sabine Baumgartner, Ronald P. Mensink, Jogchum Plat

**Affiliations:** Department of Human Biology, NUTRIM School of Nutrition and Translational Research in Metabolism, Maastricht University Medical Center, Maastricht, the Netherlands; Universita degli Studi di Milano, ITALY

## Abstract

**Background:**

Evidence is increasing that plant sterols and stanols not only lower fasting serum low-density lipoprotein concentrations, but also those of triglycerides (TG). Insight into effects of these components on postprandial TG metabolism, an emerging risk factor for cardiovascular disease, is missing.

**Objective:**

Our objective was to examine the 8-hour postprandial response after consuming plant sterol or stanol enriched margarine as part of a mixed meal.

**Methods:**

This postprandial study was part of a randomized crossover study in which 42 subjects consumed plant sterol enriched (3 g/d plant sterols), plant stanol enriched (3 g/d plant stanols), and control margarines for 4 weeks. After each period, subjects consumed a shake enriched with 3g plant sterols (sterol period), 3g plant stanols (stanol period) or no addition (control period). Subjects received a second shake with no addition after 4 hours.

**Results:**

TG and apoB48 incremental areas under the curves (iAUC) of the total (0-8h) and 1^st^ meal response (0-4h) were comparable between the meals and in all age categories (I:18-35y, II:36-52y, III:53-69y). In subjects aged 53-69y, TG iAUC after the 2^nd^ meal (4-8h) was higher in the stanol period as compared with the sterol (63.1±53.0 mmol/L/min; *P* < 0.01) and the control period (43.2±52.4 mmol/L/min; *P* < 0.05). ApoB48 iAUC after the 2^nd^ meal was higher after the stanol than after the sterol period (67.1±77.0 mg/L/min; *P* < 0.05) and tended to be higher than after the control period (43.1±64.5 mg/L/min; *P* = 0.08) in subjects aged 53-69y. These increased postprandial responses may be due to reduced lipoprotein lipase activity, since postprandial apoCIII/II ratios were increased after stanol consumption compared with the control meal.

**Conclusion:**

Postprandial TG and apoB48 responses are age-dependently increased after plant stanol consumption, which might be related to a changed clearance of triglyceride-rich lipoproteins.

**Trial Registration:**

ClinicalTrials.gov NCT01559428

## Introduction

It has unequivocally been established that daily consumption of plant sterol- and plant stanol enriched products reduces fasting serum low-density lipoprotein cholesterol (LDL-C) concentrations [1]. Functional foods enriched with plant sterols and plant stanols, as part of a healthy diet, have therefore been advocated in the management of mild hypercholesterolemia [2, 3]. Except for LDL-C, more recent studies have also suggested that plant sterols and plant stanols lower serum triglyceride (TG) concentrations [4, 5]. Prospective and case-control studies have identified high fasting serum TG concentrations as an independent risk marker for cardiovascular disease (CVD), which are routinely measured to assess CVD risk [6]. However, most people are in a non-fasting state for most part of the day and non-fasting serum TG concentrations are an emerging marker contributing to CVD risk due to the postprandial atherogenicity of TG-rich (remnant) lipoprotein particles [6, 7]. Indeed, independent of other risk markers, non-fasting TG concentrations estimated CVD risk more precisely than fasting TG concentrations did [8, 9]. Besides non-fasting TG concentrations, remnant cholesterol (cholesterol present in TG-rich particles during the fasting and non-fasting state) may be an important CVD risk marker [3, 10], illustrating the importance of including postprandial lipoprotein metabolism to assess CVD risk. Therefore, evaluation of the effects of plant sterol and plant stanol consumption on postprandial lipid metabolism is warranted. So far, only three studies have addressed postprandial lipid and lipoprotein responses after plant stanol ester intake [11–13] and 1 study assessed TG lowering after plant sterol esters [14]. Demonty et al. assessed postprandial TG concentrations 4 hours after plant sterol consumption and found lower TG concentrations after a combined intake of fish-oil and plant sterols than after an intake of fish oil alone, suggesting a contribution of plant sterols to the TG-lowering effect of fish oil [14]. The postprandial plant stanol studies have their limitations with respect to assessing postprandial responses, but they do suggest that chronic or acute consumption of plant stanol esters does not affect postprandial lipid and lipoprotein concentrations. This is remarkable, since it is generally acknowledged that fasting lipoprotein concentrations are affected by plant stanol and sterol ester consumption through inhibition of intestinal cholesterol absorption [15]. The mechanism underlying the reduction in fasting serum TGs is not known, but was suggested to be due to a reduced intestinal fatty acid absorption, at least in mice [16]. This illustrates that there is a need for a more detailed knowledge regarding postprandial effects of plant sterol and stanol ester consumption on human lipoprotein metabolism. Therefore, the objective of the present study was to examine the 8-hour postprandial response after consuming plant sterol or plant stanol ester enriched margarines as part of a shake. Since the appearance of dietary cholesterol in the circulation is delayed [17–19], we decided to provide a second mixed meal after 4 hours. This postprandial meal test was performed after completing a 4-week intervention period during which subjects consumed plant sterol or plant stanol ester enriched margarine on a daily basis.

## Subjects and Methods

### Subjects

The protocol and supporting CONSORT checklist are available as supporting information (S1 CONSORT Checklist and S1 Protocol). Details of the study have been described previously, when the effects of the intervention on fasting lipoprotein and oxyphytosterol concentrations were reported [20]. Briefly, subjects were recruited in Maastricht and met the following criteria: 18–70 years of age; body mass index (BMI) between 20–30 kg/m^2^; no active cardiovascular disease or severe medical period during the past 5 years that might interfere with the study and no use of lipid-lowering medication or a medically prescribed diet. In addition, serum total cholesterol concentrations were <7.8 mmol/L; serum TG concentrations <3.0 mmol/L and plasma glucose concentrations <6.1 mmol/L, as determined during two screening visits. All participants gave their written informed consent before entering the study. The medical ethical committee of the Maastricht University Medical Centre+ (MUMC+) had approved the protocol and the trial is registered at clinicaltrials.gov as NCT01559428.

### Diet and design

The complete 20 weeks intervention study was a randomized placebo-controlled crossover trial, which consisted of three intervention periods of 4 weeks, separated by washout periods of 4 weeks [20]. They were allocated to the intervention periods in a randomized order, based upon a computer-generated table with random numbers. Subjects replaced their own spread with the test margarine of which 20 gram had to be consumed on a daily basis, divided over at least 2 intakes. The margarines provided no, or 3.0 gram of plant sterols or stanols per day. At the end of each of the three intervention periods a postprandial test was carried out. Subjects were asked not to perform any strenuous exercise, not to consume alcohol, and to refrain from high-fat foods on the day prior to the postprandial test days. Subjects arrived at the university by public transport or by car after a 12-hour overnight fast. An intravenous cannula was placed in an antecubital vein and a fasting blood sample was collected (T = 0 min). Subjects were then asked to consume the test meal within 10 minutes. The test meal was provided as a shake and contained no, or 3.0 gram of plant sterols or stanols provided as their fatty acid esters, similar to the intervention of the preceding 4-week intervention period. The plant sterol ester mixtures contained sitosterol ester (43%), campesterol ester (25%) and stigmasterol ester (20%). Plant stanol mixtures were obtained by saturation, resulting in sitostanol ester (76%) and campestanol ester (22%). This breakfast shake contained 250 mg cholesterol and in addition 100.000 IU oil-soluble vitamin A (retinyl palmitate). Subsequent blood samples were drawn after 15, 30, 45, 60, 90, 120, 180, 240, 300, 360, 420 and 480 minutes. Following the blood sample after 240 minutes (approximately around lunch time), subjects received a second meal, which consisted again of a shake. This lunch shake contained no cholesterol, no plant sterols or stanols and no vitamin A. This second meal was provided to assess a more physiological response to a mixed meal enriched with plant sterols or plant stanols, since the appearance of dietary cholesterol (and most likely also the appearance of plant sterols and stanols) in the circulation is delayed [17–19]. The composition of the two shakes was similar for all subjects, as shown in Table 1. The shakes provided all three macronutrients to induce a postprandial response that represent the postprandial response of a meal consumed in daily life as described previously [21]. Subjects were not allowed to eat anything else, but were allowed to drink water (±500 mL) throughout the entire test day.

**Table 1:** Composition of the breakfast shakes enriched with plant sterol or stanol esters and the lunch shake.

	Breakfast shake; sterol/stanol period	Breakfast shake; control period	Lunch shake
Energy (MJ; kcal)	3.15; 755	3.14; 754	3.16; 754
Fat (en%)	59.6	59.5	60.1
SAFA (en%)	24.7	27.8	23.4
MUFA (en%)	20.9	18.8	22.2
PUFA (en%)	10.1	9.1	12.2
Protein (en%)	6.2	6.2	4.2
Carbohydrate (en%)	35.2	35.3	36.4
Cholesterol (mg)	250.2	250.2	17.3
Plant sterols or stanols (g)	3.0	-	-
Retinyl palmitate (IU)	100.000	100.000	-

Values are means ± SD. All subjects (n = 42) received the three breakfast shakes in random order. SAFA: saturated fatty acids, MUFA: monounsaturated fatty acids, PUFA: polyunsaturated fatty acids.

### Blood sampling and analyses

A clotting tube and a sodium fluoride (NaF) tube (Becton, Dickinson and Company, Franklin Lakes, NY, USA) were sampled at each time point. Serum was obtained from the clotting tube by low-speed centrifugation at 1300x g for 15 min at room temperature, at least half an hour after venipuncture and was used for the analysis of TG, apolipoprotein (apo) B48, apoCII, apoCIII and insulin concentrations. Plasma was obtained by low-speed centrifugation of the NaF tube at 1300x g for 15 min at 4°C and was used for the analysis of plasma glucose and retinyl palmitate concentrations. All serum and plasma samples were snap frozen in liquid nitrogen and stored at −80°C until analysis.

Plasma glucose concentrations (Roche Diagnostic Systems, Hoffmann-La Roche) and serum TG concentrations (GPO Trinder; Sigma-Aldrich) with correction for free glycerol were measured at baseline and after 15, 30, 45, 60 and 90 minutes, and at hourly intervals up to 8 hours. Serum insulin concentrations were measured at baseline and after 30, 45, 60 and 90 minutes and at hourly intervals up to 8 hours with a human insulin-specific RIA kit (Linco Research). The degree of insulin resistance was estimated by HOMA_IR_ as described [22]. Serum apoB48 concentrations were measured at baseline and after 30 minutes and at hourly intervals up to 8 hours with a sandwich ELISA kit containing a specific anti-apoB 48 antibody, as described previously [23] (Shibayagi). Serum apoCII and apoCIII were measured at baseline and at two-hourly intervals up to 8 hours with an ELISA kit (Randox). Retinyl palmitate concentrations were measured at baseline and after 15, 30, 45, 60 and 90 minutes and at hourly intervals up to 8 hours with high-performance liquid chromatography in a randomly selected subgroup of 13 subjects (4 men, 9 women). All samples from one subject were analyzed within one run at the end of the study.

### Statistics

All data are presented as means ± standard deviations (SD) or as means ± standard error of the mean (SEM). To evaluate the overall postprandial responses, the incremental areas under the curve (iAUC) were calculated using the trapezoidal rule as described [24]. We assessed the total postprandial response (0–8 hours), as well as the first meal response (0–4 hours) and second meal response (4–8 hours). Maximal changes were calculated by subtracting fasting concentrations (T = 0) from the maximal concentrations.

A Shapiro-Wilk normality test was performed to assess whether baseline characteristics, fasting concentrations and postprandial parameters (iAUC and maximal changes) followed a normal distribution. In addition, Mauchly’s test for sphericity was performed to examine whether the assumption of sphericity was met for fasting concentrations, iAUC’s and maximal changes between the test meals. If the assumption for sphericity was violated, the Greenhouse-Geisser correction was applied. Baseline characteristics between age categories were statistically evaluated by one-way analysis of variance (ANOVA) for normally distributed data or by a Kruskal-Wallis test for not normally distributed data. Differences in fasting concentrations, iAUC and maximal changes between the test meals were evaluated by repeated-measures ANOVA with diet as within subject factor for normally distributed data or by Friedman’s test for not normally distributed data. We also evaluated whether the observed postprandial changes were dependent on gender, body mass index (BMI) or age. There were no gender or BMI effects present. However, the interaction term diet*age was significant for TG iAUC (total and 2^nd^ meal responses), for glucose iAUC (total response), but not for apoB48 iAUC. This indicated that dietary effects were influenced by age and diet effects were therefore further evaluated per age category. Age categories were based on the age range (18–69 years) equally divided over 3 categories (category I: 18-35y (n = 17), category II: 36-52y (n = 11) and category III: 53-69y (n = 14)). Pearson correlation coefficients were used to examine linear relationships between parameters. Results were considered to be statistically significant if *P* < 0.05. All statistical analyses were performed using SPSS 18.0 for Mac Os X and adjusted by Bonferroni’s correction for multiple comparisons or by post-hoc analysis for Friedman’s test when appropriate (SPSS Inc., Chicago, IL, USA).

## Results

**Fig 1:**
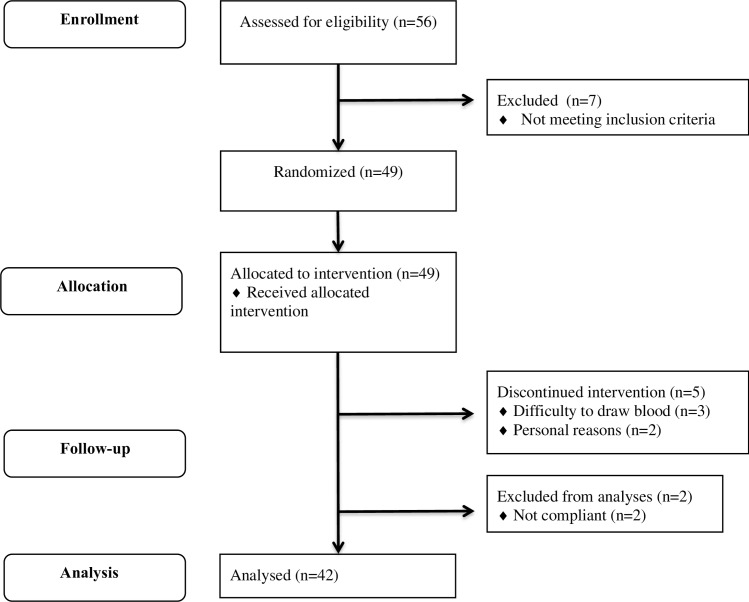
Subject flow chart.

A flow diagram of subjects throughout the study is shown in Fig 1. Fifty-six subjects were assessed for eligibility and seven subjects were excluded because they did not met pre-defined inclusion criteria. Therefore, 49 subjects were randomly allocated to receive the intervention treatment. Two subjects discontinued the intervention because of personal reasons and for three subjects it was too difficult to draw blood. Further, two subjects were excluded from the analyses because they were not compliant with the requested dietary intake. A total of 42 subjects completed the study and were included in the analyses. Baseline characteristics of study participants are shown in Table 2 and characteristics per age category are displayed in S1 Table.

**Table 2:** Baseline characteristics of subjects.

	All subjects (n = 42)
Age (y)	42 ± 18
Male / female (n)	17 / 25
Weight (kg)	73.5 ± 11.3
Height (m)	1.72 ± 0.09
BMI (kg/m^2^)	24.7 ± 2.8
Glucose (mmol/L)	5.26 ± 0.55
HOMA-index	1.42 ± 0.65
TCH (mmol/L)	5.73 ± 1.13
HDL-C (mmol/L)	1.69 ± 0.38
TC/HDL ratio	3.52 ± 1.03
TG (mmol/L)	1.13 ± 0.46

### Postprandial lipemia

**Fig 2:**
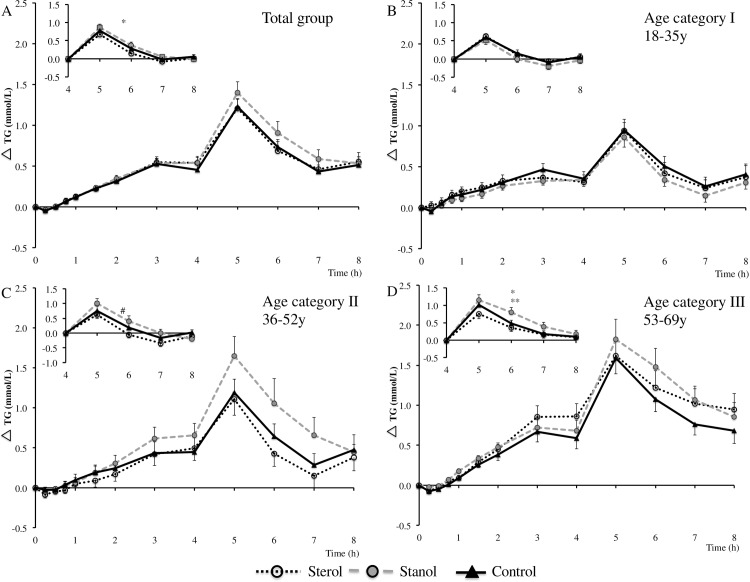
Serum triglyceride concentration differences after consumption of a mixed meal containing no, or 3.0 gram of plant sterols or plant stanols. (A) total group: 18–69 years (n = 42), significant difference iAUC^2^ between stanol and sterol period *(*P* < 0.05); (B) age category I: 18–35 y (n = 17); (C) age category II: 36–52 y (n = 11), trend difference iAUC^2^ between stanol and sterol period ^#^(*P* = 0.07); (D) age category III: 53–69 y (n = 14), significant difference iAUC^2^ between stanol and control period *(*P* < 0.05) and stanol and sterol period **(*P* < 0.01). iAUC^2^ in age category I was tested by Friedman’s test for not normally distributed data. Inserts show second meal responses (4–8 hours). Data are means ± SEM. iAUC^2^: incremental AUC after the 2^nd^ meal (4-8h).

Fasting serum TG and apoB48 concentrations were not different after the three intervention periods. The postprandial TG time curves of the total group and the different age categories are presented in Fig 2, while Table 3 shows fasting and AUC data of the total group (fasting and AUC data of the age categories is presented in S2 Table). Serum TG concentrations gradually increased after consumption of the first meal to peak at 3–4 hours. A second peak was observed already one hour after consumption of the second meal (after 5 hours). In the total group, TG iAUC of the total TG response (iAUC^T^) and of the 1^st^ meal response (iAUC^1^) were comparable between the test meals, while a higher iAUC after the 2^nd^ meal (iAUC^2^) was observed after consumption of the stanol meal compared with the sterol meal (difference of 28.3 ± 63.1 mmol/L/min; *P* < 0.05). In the first age category (18–35 y),iAUC^T^, iAUC^1^ and iAUC^2^ were comparable between the test meals and in the second age category (36–52 y), iAUC^T^ and iAUC^1^ were comparable, but there was a trend for a higher iAUC^2^ in the stanol period as compared with the sterol period (difference of 46.8 ± 58.8 mmol/L/min; *P =* 0.07). In the third age category (53–69 y), iAUC^T^ and iAUC^1^ were again comparable between the test meals, but there was a significantly higher iAUC^2^ in the stanol period as compared with the sterol period (63.1 ± 53.0 mmol/L/min; *P* < 0.01) and the control period (43.2 ± 52.4 mmol/L/min; *P* < 0.05).

**Table 3:** Fasting concentrations, iAUC and maximal increase from baseline in TG and ApoB48 concentrations after consumption of a mixed meal containing no, or 3.0 gram of plant sterols or plant stanols.

	Control period	Sterol period	Stanol period
TG fasting (mmol/L)	1.08 ± 0.57	1.11 ± 0.43	1.04 ± 0.42
TG iAUC^T^ (mmol/L/min)	241.3 ± 141.4	257.7 ± 182.9	282.0 ± 191.7
TG iAUC^1^ (mmol/L/min)	71.0 ± 39.8	79.4 ± 51.7	77.2 ± 48.2
TG iAUC^2^ (mmol/L/min)	75.6 ± 56.6	64.3 ± 49.0	92.6 ± 71.0 ^a^
MaxTG (mmol/L)	1.23 ± 0.62	1.30 ± 0.67	1.46 ± 0.87 ^b^
ApoB48 fasting (mg/L)	12.7 ± 8.8	11.5 ± 6.3	11.5 ± 7.4
ApoB48 iAUC^T^ (mg/L/min)	3148.3 ± 1808.7	3061.2 ± 2031.2	3307.0 ± 1738.7
ApoB48 iAUC^1^ (mg/L/min)	1096.7 ± 546.1	1059.6 ± 568.7	1076.8 ± 588.8
ApoB48 iAUC^2^ (mg/L/min)	1192.5 ± 823.9	1080.3 ± 902.7	1403.2 ± 927.7^c^
MaxApoB48 (mg/L)	13.2 ± 6.7	13.2 ± 7.8	14.2 ± 7.0

Data are means ± SD. iAUC^T^: incremental AUC of the total response, iAUC^1^: incremental AUC after the 1^st^ meal (0-4h), iAUC^2^: incremental AUC after the 2^nd^ meal (4-8h).

^a^ significant difference stanol period compared with sterol period (*P* = 0.018)

^b^ trend for difference stanol period compared with control period (*P* = 0.09)

^c^ trend for difference stanol period compared with sterol period (*P* = 0.06) and control period (*P* = 0.09).

Age was positively correlated with iAUC^T^ in all three intervention periods (control: r = 0.47; *P* < 0.01, sterol: r = 0.44; *P* < 0.01, stanol: r = 0.56; *P* < 0.01) and with iAUC^1^ (control: r = 0.27; *P* = 0.08 (trend), sterol: r = 0.31; *P* < 0.05, stanol: r = 0.42; *P* < 0.01), while the correlation between age and iAUC^2^ was only significant in the stanol period (r = 0.57; *P* < 0.01). The maximal TG (maxTG) concentration increased with age in all three periods (control: r = 0.49; *P* < 0.01, sterol: r = 0.36; *P* < 0.05, stanol: r = 0.45; *P* < 0.01). In addition, there was a trend towards a higher maxTG after stanol consumption compared with sterol consumption in age category II (0.60 ± 0.74 mmol/L; *P* = 0.07).

**Fig 3:**
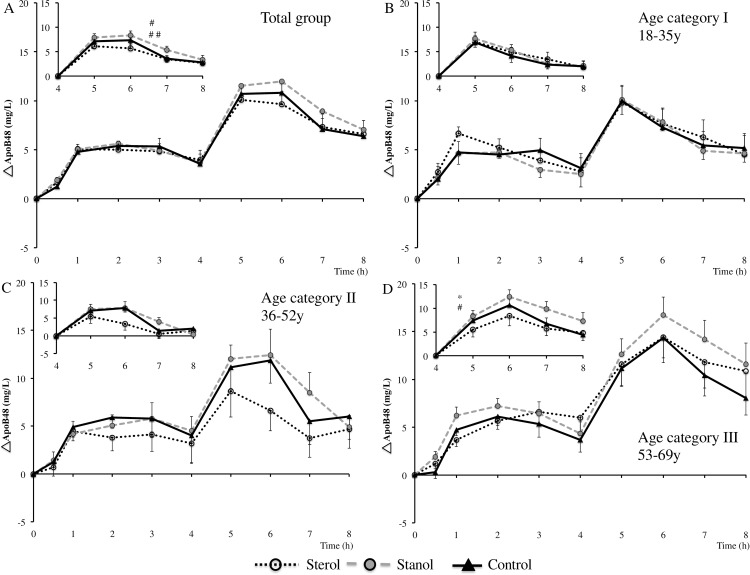
Serum apoB48 concentration differences after consumption of a mixed meal containing no, or 3.0 gram of plant sterols or plant stanols. (A) total group: 18–69 y (n = 42), trend difference iAUC^2^ between stanol and sterol ^#^(*P* = 0.06) and between stanol and control ^##^(*P* = 0.09); (B) age category I: 18–35 y (n = 17); (C) age category II: 36–52 y (n = 11); (D) age category III: 53–69 y (n = 14), significant difference iAUC^2^ between stanol and sterol period *(*P* < 0.05) and trend difference iAUC^2^ between stanol and control period ^#^(*P* = 0.08). iAUC^T^ and maxApoB48 in age category III were tested by Friedman’s test for not normally distributed data. Greenhouse-Geisser correction was applied for iAUC^1^ in age category III. Inserts show second meal responses (4–8 hours). Data are means ± SEM. iAUC^2^: incremental AUC after the 2^nd^ meal (4-8h).

The postprandial ApoB48 time curves of the total group and the different age categories are presented in Fig 3, while Table 3 shows fasting and AUC data of the total group (fasting and AUC data of the age categories is presented in S3 Table). Interestingly, peak apoB48 concentrations were earlier reached (1 hour) after the first meal as compared with the peak TG concentration (3 hours). Another clear difference is that the apoB48 time-to-peak after the second meal was delayed from the younger to the older age categories (i.e. from 5 hours to 6 hours after meal ingestion), which was not seen in the postprandial TG response. ApoB48 iAUC^T^ and iAUC^1^ were comparable between the three test meals in the total group and in all age categories. There was a trend for a higher apoB48 iAUC^2^ after the stanol period in the total group compared with the sterol period (32.3 ± 87.3 mg/L/min; *P* = 0.06) and the control period (21.1 ± 61.0 mg/L/min; *P* = 0.09). When separated for age categories, iAUC^2^ was comparable in the first two age categories, but in age category III the iAUC^2^ after stanol consumption was increased as compared with the sterol period (67.1 ± 77.0 mg/L/min; *P* < 0.05) and tended to be higher after stanol consumption compared with the control period (43.1 ± 64.5 mg/L/min; *P* = 0.08).

Age was positively correlated with iAUC^T^ in the stanol period (r = 0.47; *P* < 0.01) and with iAUC^2^ in the control and stanol period (r = 0.32; *P* < 0.05 and r = 0.43; *P* < 0.01, respectively). The maximal apoB48 (maxApoB48) concentration correlated with age in the stanol period (r = 0.43; *P* < 0.01), but not in the sterol and control period. In addition, maxApoB48 concentrations were higher after stanol consumption compared with the control and sterol period in age category III (2.9 ± 4.2 and 1.8 ± 5.9 mg/L, respectively; *P* < 0.05).

Table 4 shows fasting and AUC data of the total group for apoCII, apoCIII and apoCIII/II values. Postprandial parameters separated for the different age categories are reported in S4 Table and corresponding postprandial time curves are shown in S1 Fig. Fasting apoCII and apoCIII values, and the apoCIII/CII ratio were not different after the three intervention periods. Postprandial apoCII and apoCIII responses were comparable in the total group and in all age categories after the three meals. For postprandial apoCIII responses, only a difference in AUC^T^ in the total group after the sterol and control period was seen (median difference -322 (range: -1618–1683; *P* <0.05). We also assessed apoCIII/CII ratio, since these two apoproteins have opposite effects on lipoprotein lipase (LPL) activity, e.g. apoCII activates LPL, whereas apoCIII inhibits LPL activity. Postprandial apoCIII/CII ratios were comparable after all three mixed meals in the total group and in the first two age categories, while the 2^nd^ meal response in age category III was increased after stanol consumption compared with the control meal (median difference 33.5 (range: -21.7–244.0); *P* < 0.05).

**Table 4:** Fasting concentrations and AUCs of ApoCII, ApoCIII and ApoCIII/II values after consumption of a mixed meal containing no, or 3.0 gram of plant sterols or plant stanols.

	Control period	Sterol period	Stanol period
ApoCII fasting (mg/dL)	3.6 (1.5–7.7)	3.4 (0.9–7.2)	3.4 (1.2–7.0)
ApoCII AUC^T^ (mg/dL/min)^a^	1760 (643–3354)	1619 (472–3260)	1580 (593–3367)
ApoCII AUC^1^ (mg/dL/min)^a^	878 (345–1714)	637 (245–1660)	817 (290–1714)
ApoCII dAUC^2^ (mg/dL/min)^a^	28.0 (0.0–116.3)	26.0 (0.0–142.0)	45.2 (0.0–315.7)
ApoCIII fasting (mg/dL)	10.3 (5.4–17.1)	9.5 (4.1–16.5)	9.5 (4.8–16.1)
ApoCIII AUC^T^ (mg/dL/min)^a^	4765 (2546–8329)	4400 (1901–7772)^b^	4432 (2292–7590)
ApoCIII AUC^1^ (mg/dL/min)^a^	2362 (1263–4087)	2225 (943–3863)	2242 (1109–3804)
ApoCIII iAUC^2^ (mg/dL/min)	69.4 (0.0–1609.6)	61.0 (0.0–422.9)	96.3 (0.0–353.1)
ApoCIII/II ratio fasting	2.7 (1.6–5.3)	2.8 (2.0–7.0)	3.0 (1.7–5.5)
ApoCIII/II iAUC^T^	26.8 (0.0–283.6)	34.4 (0.0–328.6)	46.6 (0.0–322.0)
ApoCIII/II iAUC^1^	0.0 (0.0–44.2)	1.9 (0.0–109.9)	0.0 (0.0–112.3)
ApoCIII/II iAUC^2^	47.0 (5.5–259.8)	45.4 (0.0–263.4)	60.8 (0.0–375.3)

All parameters were tested by Friedman’s test for not normally distributed data and are presented as medians (ranges). AUC^T^: AUC of the total apoC response, AUC^1^: AUC after the 1^st^ meal (0-4h), iAUC^2^: incremental AUC after the 2^nd^ meal (4-8h), dAUC^2^: decremental AUC after the 2^nd^ meal.

^a^ Responses are given in AUC or dAUC since time curves fluctuate below and above baseline levels

^b^ Significant difference sterol period compared with control period (*P* = 0.04).

**Fig 4:**
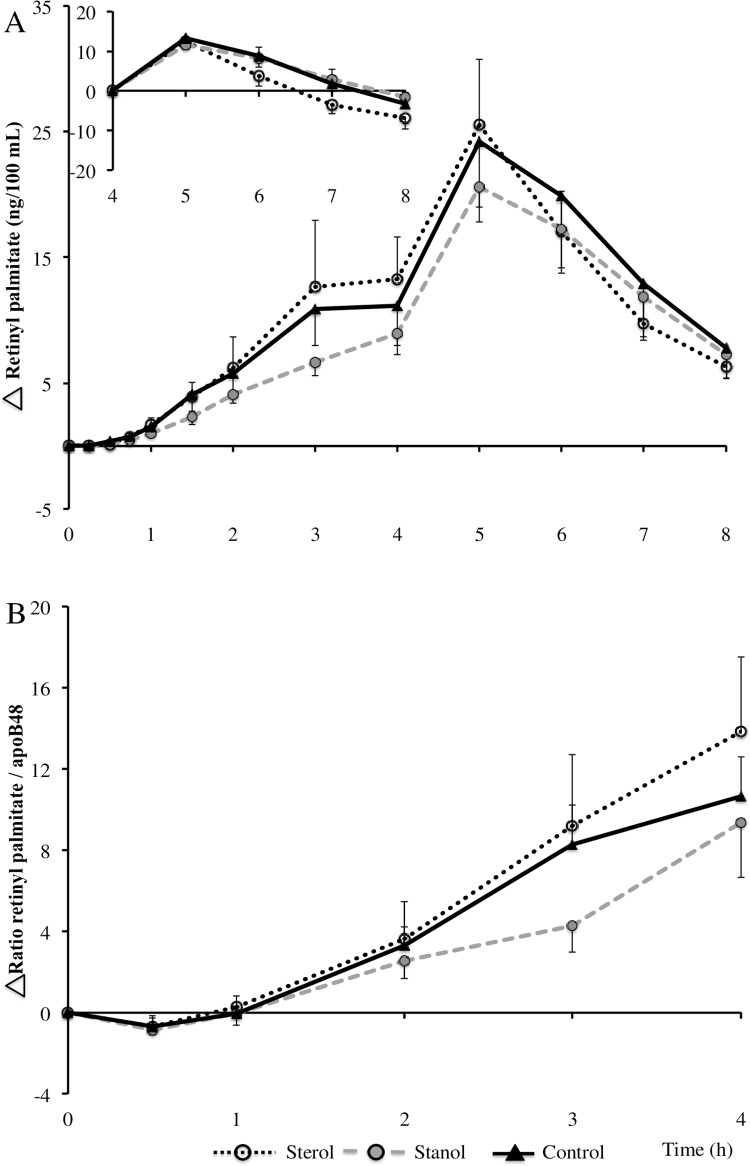
**(A) Plasma retinyl palmitate concentration differences and (B) ratio difference retinyl palmitate/apoB48 (n = 13) after consumption of a mixed meal containing no, or 3.0 gram of plant sterols or plant stanols.** Insert shows second meal response (4–8 hours). Data are means ± SEM.

Retinyl palmitate was incorporated only in the breakfast shake and appearance in the circulation provides information about the lipid content of the chylomicron fraction, whereas apoB48 provides information about the number of particles [25, 26]. Postprandial plasma retinyl palmitate concentrations (shown in Fig 4, panel A) were determined in 13 subjects and the curve closely resembled the serum TG curve, showing a first meal peak between 3–4 hours and a second meal peak one hour after consuming the lunch shake. Due to the smaller sample size (n = 13 instead of n = 42), no statistical analyses in age subgroups were performed on the retinyl palmitate data. Postprandial TG curves were comparable in the subgroup of 13 subjects compared with the entire group of 42 subjects. Changes in postprandial TG concentrations always consist of a combined effect of changes in chylomicron and very low-density lipoprotein (VLDL) particle numbers and composition. To visualize the postprandial lipid response of the first 4 hours, we plotted the retinyl palmitate/apoB48 ratio (Fig 4, panel B). This ratio provides information about the amount of TG derived from the diet as compared to the number of chylomicron particles in the circulation. More specifically, it provides information about the lipid content of chylomicrons, e.g. their size [27]. Fig 2A–2C show increases in apoB48 concentrations up to 1 hour, after which concentrations remained relatively stable up to 4 hours, indicating that production rate of chylomicrons equals their clearance rate. This observation, combined with Fig 4B, suggests that TG-poor chylomicrons are produced during the first hour, after which the dietary TG content of chylomicrons (e.g. size) increases up to 4 hours. No diet effects were observed for retinyl palmitate/apoB48 ratios.

### Postprandial glycemia

**Fig 5:**
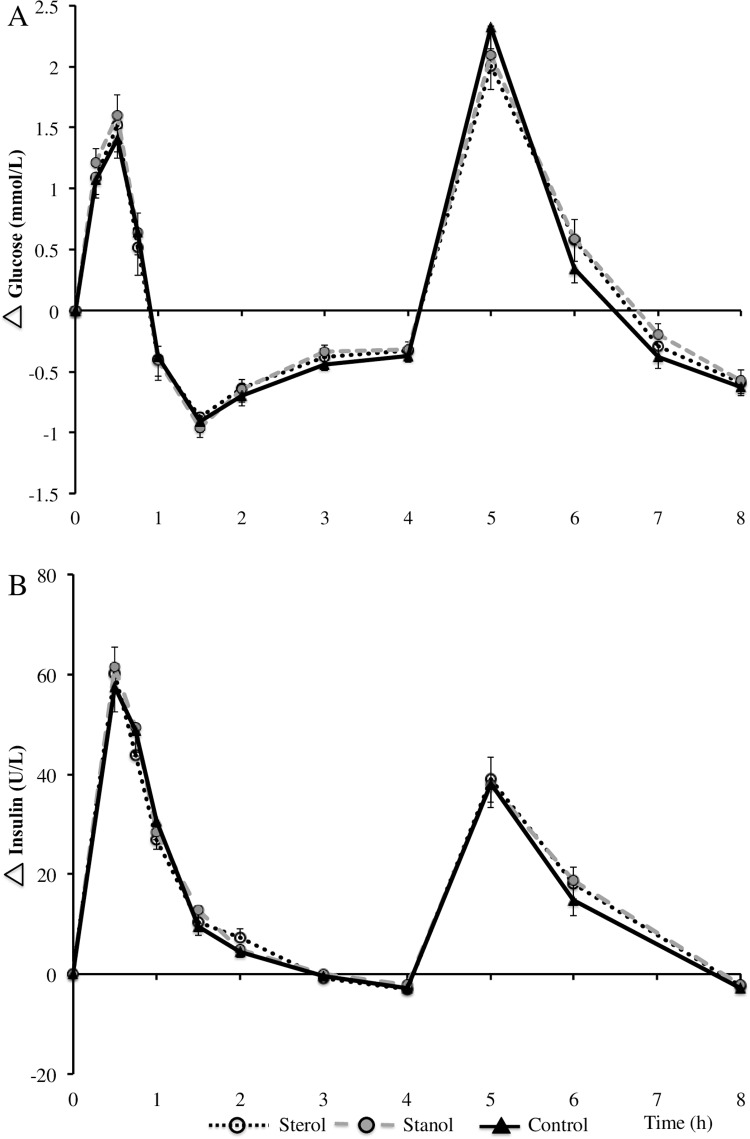
**(A) Plasma glucose and (B) insulin concentration differences (n = 42) after consumption of a mixed meal containing no, or 3.0 gram of plant sterols or plant stanols.** Data are means ± SEM.

Fasting glucose and insulin concentrations were comparable between the three test days. Postprandial plasma glucose concentrations increased during the first 30 min after consumption of the first meal, returned to baseline after 60 min and remained below baseline till consumption of the second meal **(**Fig 5**).** Plasma glucose concentrations were maximally increased one hour after consumption of the second meal. Glucose iAUC^T^ was higher in the stanol period compared with the sterol period in age category I (229.4 ± 108.1 vs. 168.5 ± 106.4 mmol/L/min; *P* < 0.05; data not shown). Postprandial glucose responses did not differ between the test meals in age category II and III. Serum insulin concentrations were maximally increased 30 min after first meal consumption and returned to baseline concentrations after 180 min. A second (smaller) peak was observed after consumption of the second meal. No diet effects were observed for postprandial insulin responses in all subjects or in the different age categories.

## Discussion

In this randomized controlled trial, we found an increased TG and apoB48 second meal response in subjects aged between 53 and 69 years after consuming a mixed meal enriched with plant stanol esters as compared to a mixed meal with or without plant sterol esters.

The LDL-C lowering effect of plant sterol and plant stanols is well known, but recent studies have suggested that plant sterol and plant stanol esters also reduce fasting TG concentrations [1, 5] by a so far unknown mechanism. This is however an interesting effect of plant sterol and stanol esters that warrants further study since both fasting and postprandial TG concentrations are positively associated with CVD risk [7–9]. However, so far only three studies so far have addressed the postprandial effects of foods enriched with plant stanol esters on lipid and lipoprotein metabolism and one study evaluated the effect of plant sterol esters on postprandial TG concentrations. Demonty et al. showed that plant sterol ester consumption contributed to the postprandial TG-lowering effect of fish-oil but it was not possible to draw conclusions regarding an independent effect of plant sterol esters. [14]. Relas et al. performed two postprandial mixed meal studies enriched with plant stanol esters: a 24-hour study with 3.0g of plant stanol esters after a 2-week intervention period and an acute study with 1.0g of plant stanol esters [12, 13]. In both studies, the addition of plant stanol esters to a high-fat breakfast did not affect postprandial cholesterol and TG concentrations in serum or in lipoprotein fractions. Gylling et al. provided a low-fat breakfast containing 4.5g of plant stanol esters after a 10-week intervention period (daily intake 8.8g of plant stanol esters) [11]. Unfortunately, changes in serum TG and apoB48 concentrations were not determined in that study. The different postprandial study designs complicates the comparison to our study, especially because we found that plant stanol esters affect the second meal TG and apoB48 response by using a sequential meal protocol with regular blood sampling. We demonstrated that iAUCs for TG and apoB48 responses were similar after the first meal, while these responses were increased after consumption of the second meal in the plant stanol period. This effect was stronger with increasing age and more evident compared to the sterol period than to the control period. These increased postprandial responses might have resulted from an increased production of triglyceride-rich lipoproteins (TRLs) or from a decreased clearance of these particles. We can only speculate which of these processes have been affected by plant stanol consumption. A recent study in mice showed reduced TG concentrations after plant sterol and stanol feeding and attributed this reduction to a decreased hepatic VLDL secretion [28], which could indicate that our increased TG response is probably not explained by a change in VLDL production. On the other hand, there are indications that a second meal response predominantly addresses effects on TG clearance [29, 30], which might suggest that postprandial TG and chylomicron particle clearance are less efficient after plant stanol consumption. After plant stanol consumption, plasma plant sterol concentrations decrease [31], which may offer an explanation for a potential change in TG clearance. LPL activity plays a crucial role in TG clearance and since LPL activity is difficult to measure, we analyzed postprandial apoCII and apoCIII concentrations. Even though measuring apoCII and apoCIII in different lipoprotein sub fractions would have provided additional information over their measurement in total serum, both are modulators of LPL activity and give some insights into TG clearance [32]. We demonstrated that the 2^nd^ meal response of apoCIII/CII ratio was increased after plant stanol consumption compared with the control meal in age category III, which suggests a reduction of LPL activity [33, 34] and thus a delayed clearance of TRL. This observation is in line with the observed increased TG and apoB48 postprandial responses in this age category and might indicate that TRL clearance is impaired due to a reduced LPL activity after plant stanol consumption in older subjects. Another explanation for the second meal effect after plant stanol ester consumption might be found in changed oxyphytosterol concentrations (oxidized plant sterols). We have already reported that plant stanol consumption reduced fasting oxyphytosterol concentrations [20]. Oxysterols (oxidized cholesterol) are natural ligands of LXR, which in turn induce LPL expression. There are some suggestions that oxyphytosterols activate LXR [35], which could lead to an induction of LPL expression. If so, a positive effect of oxyphytosterols on LPL activity might be lost after plant stanol consumption, which could have resulted in decreased TG clearance.

The increase in TG concentrations after consumption of a second meal seems to contradict recent findings of reduced fasting TGs concentrations after plant stanol consumption [4, 5]. However, reductions in fasting TG concentrations were only observed in subjects with elevated fasting TG concentrations and there is no data describing the effect of plant sterol and plant stanol consumption on postprandial responses in these populations. In the present study in apparently healthy, slightly hypercholesterolemic, normotriglyceridemic subjects, no effects of plant sterol and stanol esters on fasting TGs were found–in all age categories–[20], while postprandial TG and apoB48 responses were increased in the plant stanol ester group. For now, it remains speculative whether the same observation on postprandial TGs would have been found in a population with elevated fasting TGs. It should however be noted that the observed plant stanol effect after consumption of a second meal was only present in older subjects, which may form a relatively large part of the target population for the consumption of plant sterol and plant stanol enriched products i.e. subjects with mild hypercholesterolemia [2].

This study also addresses the difference in conclusions that could be made after single meal consumption or after including a second meal. Our results highlight the metabolic consequences of sequential meal consumption in general. Retinyl palmitate was added to the first breakfast meal. After absorption, retinyl palmitate associates with chylomicrons and their remnants until taken up—without being re-secreted—by the liver, providing information regarding the lipid content of chylomicrons [26, 36]. In contrast, apoB48 concentrations provide information regarding the number of chylomicron particles [25]. Even though retinyl palmitate has several drawbacks, i.e. mainly accurate in early postprandial period (0-9h) [26], it provides clear evidence for a second meal effect. Retinyl palmitate peak peaked one hour after consumption of the lunch shake (while no retinyl palmitate was present in the lunch shake), which indicates that this second meal induced chylomicron appearance contains fat from the previous breakfast shake. This illustrates the importance of choosing the appropriate study design to address a certain research question, especially when investigating nutritional effects on postprandial lipid responses. In addition, we calculated ratios of retinyl palmitate (a marker for dietary TG) concentrations to apoB48 concentrations and proposed that TG-enrichment of chylomicrons continues to increase up to 4 hours without a concomitant increase in apoB48 concentrations after the first hour. This suggests that more TG-rich chylomicrons are produced, which agrees with earlier findings suggesting that dietary lipid transport following a meal is mainly through increased chylomicron particle size and less to an increase in particle numbers, i.e. an increase in lipid content per chylomicron [37].

In conclusion, this study demonstrates an age-dependent increase in the postprandial TG and apoB48 response after plant stanol ester consumption as part of a mixed meal, compared to a control meal and to a meal enriched in plant sterol esters. Further research is warranted to confirm and extend these results. Also, the mechanism by which plant sterols and plant stanols possibly exert different effects on postprandial lipemic responses needs to be investigated, and if these effects have health consequences. In addition, our findings highlight the importance of studying second meal responses, as it provides more detailed information on postprandial lipid and lipoprotein responses.

## Supporting Information

S1 CONSORT Checklist (DOC)

S1 Fig Serum apoCII and CIII median differences and apoCIII/II ratio differences after consumption of a mixed meal containing no, or 3.0 gram of plant sterols or plant stanols. (A) serum apoCII concentration differences in age category I: 18–35 years; (B) age category II: 36–52 years; (C) age category III: 53–69 years. (D) serum apoCIII concentration differences in age category I: 18–35 years; (E) age category II: 36–52 years; (F) age category III: 53–69 years. (G) apoCIII/CII ratio differences in age category I: 18–35 years; (H) age category II: 36–52 years; (I) age category III: 53–69 years, significant difference iAUC^2^ between stanol and control period *(*P* < 0.05). Inserts show second meal responses (4–8 hours). iAUC^2^: incremental AUC after the 2^nd^ meal (4-8h).
(TIF)

S1 Protocol (PDF)

S1 Table Baseline characteristics of subjects separated per age category. (DOCX)

S2 Table Fasting concentrations, iAUC and maximal increases from baseline in TG concentrations after consumption of a mixed meal containing no, or 3.0 gram of plant sterols or plant stanols separated per age category. (DOCX)

S3 Table Fasting concentrations, iAUCs and maximal increases from baseline in apoB48 concentrations after consumption of a mixed meal containing no, or 3.0 gram of plant sterols or plant stanols separated per age category. (DOCX)

S4 Table Fasting concentrations and AUCs of ApoCII, ApoCIII and ApoCIII/II values after consumption of a mixed meal containing no, or 3.0 gram of plant sterols or plant stanols separated per age category. (DOCX)
