# Supplementary figures and images for: Effects of a Plant Sterol or Stanol Enriched Mixed Meal on Postprandial Lipid Metabolism in Healthy Subjects
Source: PLoS One. 2016 Sep 9;11(9):e0160396. doi: 10.1371/journal.pone.0160396 (PMC5017646; doi:10.1371/journal.pone.0160396)

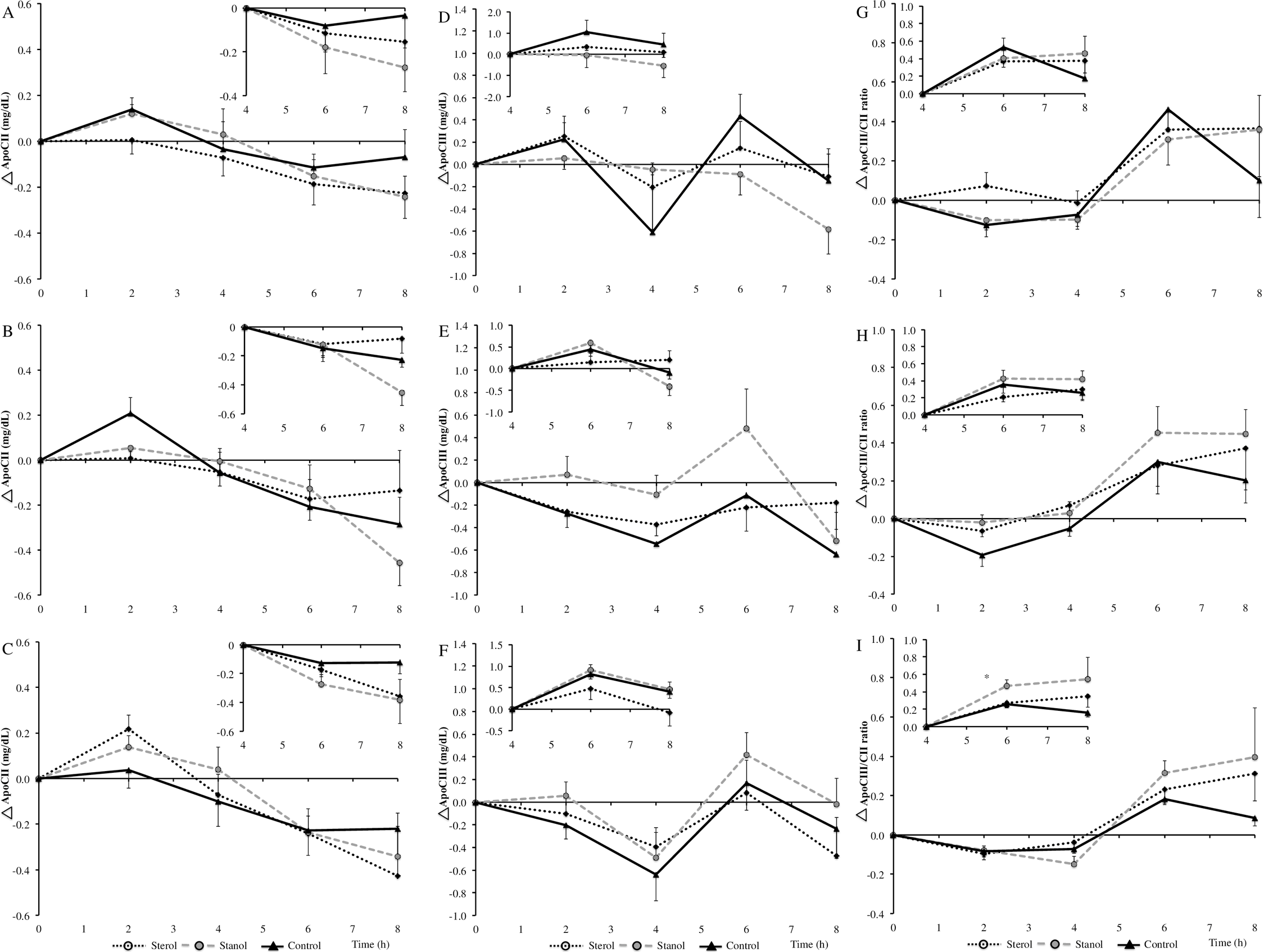

Supplement: S1 Fig — (A) serum apoCII concentration differences in age category I: 18–35 years; (B) age category II: 36–52 years; (C) age category III: 53–69 years. (D) serum apoCIII concentration differences in age category I: 18–35 years; (E) age category II: 36–52 years; (F) age category III: 53–69 years. (G) apoCIII/CII ratio differences in age category I: 18–35 years; (H) age category II: 36–52 years; (I) age category III: 53–69 years, significant difference iAUC2 between stanol and control period *(P < 0.05). Inserts show second meal responses (4–8 hours). iAUC2: incremental AUC after the 2nd meal (4-8h). (TIF) [file pone.0160396.s002.tif]
