# Supplementary material for: Effects of a Plant Sterol or Stanol Enriched Mixed Meal on Postprandial Lipid Metabolism in Healthy Subjects
Source: PLoS One. 2016 Sep 9;11(9):e0160396. doi: 10.1371/journal.pone.0160396 (PMC5017646; doi:10.1371/journal.pone.0160396)
Supplement: S1 Protocol — (PDF) [file pone.0160396.s003.pdf]

The effects of plant sterol and stanol esters on serum oxyphytosterol concentrations in healthy human subjects

De effecten van plantaardige sterolen en stanolen op de oxyphytosterolen concentraties in het bloed van gezonde mensen

Written by:

Sabine Baumgartner

Ronald P. Mensink

Jogchum Plat

Department of Human Biology

Nutrition and Toxicology Research Institute Maastricht

Maastricht University

P.O. Box 616

6200 MD Maastricht

The Netherlands

Telephone: +31-43-3881309

FAX: +31-43-3670976

E-mail: J.Plat@HB.unimaas.nl

Version number 2; 05-02-2010

PROTOCOL TITLE

The effects of plant sterol and stanol esters on serum oxyphytosterol concentrations in healthy human subjects

De effecten van plantaardige sterolen en stanolen op de oxyphytosterolen concentraties in het bloed van gezonde mensen

Protocol ID	PRJIP07_RAISIO_METC
Short title	Phytosterols and oxyphytosterol concentrations
Version	2.0
Date	05-02-2010
Coordinating investigator	Sabine Baumgartner, MSc<br>Department of Human Biology<br>School for Nutrition, Toxicology and Metabolism<br>Maastricht University<br>P.O. Box 616<br>6200 MD Maastricht<br>The Netherlands<br><br>Telephone: +31-43-3881313<br>Fax: +31-43-3670976<br>E-mail: sabine,baumgartner@HB.unimaas.nl
Principal investigators	Ronald P. Mensink, PhD<br>Jogchum Plat, PhD
Independent physician(s)	H. Kuipers, MD<br>Department of Human Biology<br>School for Nutrition, Toxicology and Metabolism<br>Maastricht University<br>P.O. Box 616<br>6200 MD Maastricht<br>The Netherlands<br>Tel. 043-3881311

Name	Signature	Date
Principal Investigator:<br>J. Plat, PhD		

Table of Contents

SUMMARY 7

1. INTRODUCTION AND RATIONALE 8
    - 1.1 Plant sterols and stanols 8
    - 1.2 Oxyphytosterols 8
    - 1.3 Previous research 9
2. OBJECTIVES 10
3. STUDY DESIGN 11
4. STUDY POPULATION 12
    - 4.1 Population 12
    - 4.2 Screening visit 12
    - 4.3 Inclusion criteria 13
    - 4.4 Exclusion criteria 13
    - 4.5 Sample size calculation 14
5. TREATMENT OF SUBJECTS 14
    - 5.1 Placebo controlled dietary intervention 14
    - 5.2 Postprandial test 14
    - 5.3 Composition of the experimental products and menus 15
6. METHODS 17
    - 6.1 Randomization, blinding and treatment allocation 17
    - 6.2 Study procedures 17
    - 6.3 Study parameters 19
    - 6.3.1 Metabolic risk markers 19
    - 6.3.2 Food intake 21
    - 6.3.3 Body weight 21
    - 6.4 Withdrawal of individual subjects 21
    - 6.5 Replacement of individual subjects after withdrawal 21
    - 6.6 Follow-up of subjects withdrawn from treatment 21
7. SAFETY REPORTING 21
    - 7.1 Section 10 WMO event 21
    - 7.2 Adverse and serious adverse events 21
    - 7.3 Follow-up of adverse events 22
8. STATISTICAL ANALYSIS 22

9. ETHICAL CONSIDERATIONS 22
9.1 Regulation statement 22
9.2 Recruitment and consent 22
9.3 Privacy 23
9.4 Benefits and risks assessment, group relatedness 23
9.5 Compensation for injury 23
9.6 Incentives 24
10. ADMINISTRATIVE ASPECTS AND PUBLICATION 25
11. REFERENCES 26
APPENDIX 1: Design of the study 28
APPENDIX 2: Composition of the investigational products 29
APPENDIX 3: Declaration of safety of the investigational products 30
APPENDIX 4: HACCP analysis 32
APPENDIX 5: Certificate of analysis of retinyl palmitate 33
APPENDIX 6: Blood analysis protocol 34

List of abbreviations and relevant definitions

ApoA-I	Apolipoprotein A-I
ApoB100	Apolipoprotein B100
ApoB48	Apolipoprotein B48
CCMO	Central Committee on Research Involving Human Subjects
Hb	Haemoglobin
HDL-C	High-density lipoprotein cholesterol
hsCRP	High-sensitive C-reactive protein
IL-6	Interleukin-6
IDL-C	Intermediate-density lipoprotein
LDL-C	Low-density lipoprotein cholesterol
MCP-1	Monocyte chemotactic protein-1
METC	Medical research ethics committee (MREC); in Dutch: medisch ethische toetsing commissie (METC)
(S)AE	(Serious) Adverse Event
sE-Selectin	Soluble E-Selectin
sICAM-1	Soluble Intercellular adhesion molecule 1
VCAM-1	Vascular cell adhesion molecule 1
Sponsor	The sponsor is the party that commissions the organisation or performance of the research, for example a pharmaceutical company, academic hospital, scientific organisation or investigator. A party that provides funding for a study but does not commission it is not regarded as the sponsor, but referred to as a subsidising party.
TAG	Triacylglycerol
TCH	Total cholesterol
TNF-α	Tumor necrosis factor alpha
TNFR	Tumor necrosis factor receptor
VLDL	Very low-density lipoprotein
vWF	Von willebrand factor
WMO	Medical Research Involving Human Subjects Act (Wet Medisch-wetenschappelijk Onderzoek met Mensen

SUMMARY

Rationale: Plant sterols and stanols (also called phytosterols and phytostanols) are structurally related to cholesterol, but absorbed to a much lesser extent. Due to this structural similarity, plant sterols and stanols inhibit intestinal cholesterol absorption and lower serum LDL cholesterol concentrations by about 10% at daily intakes of 2.5 g. Plant sterol- and stanol-enriched food products are therefore widely available on the market to lower the risk for coronary heart disease. Like cholesterol, plant sterols undergo however oxidation, which results in the formation of oxyphytosterols. Animal studies have now suggested that oxyphytosterols are atherogenic. Although oxyphytosterols have been identified in human serum samples, the effect of an increased intake of plant sterols on serum oxyphytosterol concentrations in humans is not known. On the other hand, plant stanols cannot be oxidized and lower not only cholesterol absorption, but also plant sterol absorption.

Objective: The major objective of the present study is to examine the effects of dietary plant sterols and stanols on fasting serum concentrations of oxyphytosterols. The minor objective is to investigate the effects of these products on postprandial serum oxyphytosterol concentrations.

Study design: A randomized, double-blind, placebo-controlled cross-over design. The total study duration will be 20 weeks, consisting of 3 test periods of 4 weeks in which the volunteers will use the investigational products. Each period will be separated by a wash-out period of 4 weeks. At the end of each test period, a postprandial test will be carried out.
Study population: 48 apparently healthy male and female subjects, aged 18-70 years.

Intervention: During each test period, the volunteers will use 20 grams per day of a plant sterol-enriched margarine (providing daily 3.0 gram of plant sterols), a plant stanol-enriched margarine (providing daily 3.0 gram of plant stanols), or a control margarine. During the 4-week wash-out period, they will return to their normal eating habits. For the postprandial test, the subjects will consume a fat rich-test meal enriched with no or with 3.0 gram plant sterols or stanols at breakfast. In addition, a fat-rich lunch will be provided. Total follow-up during the postprandial period is 8 hours.

Main study parameters/endpoints: Blood samples will be drawn at the beginning and end of each test period, and during the postprandial test. The samples will be analyzed for serum / plasma concentrations of plant sterols and oxyphytosterols, lipoproteins, glucose, insulin, and for markers reflecting low-grade systemic inflammation and endothelial dysfunction.

Nature and extent of the burden and risks associated with participation, benefit and group relatedness: Blood samples will be drawn on 9 different occasions in a time frame of 20 weeks with a total amount of 514.5 mL. During the screening procedure 11 mL blood will be sampled. Furthermore, subjects will be asked to fill out a food frequency questionnaire

three times at the end of each experimental period. Apart from a haematoma or bruise, which can occur during or after venipuncture, no side effects are expected.

1. INTRODUCTION AND RATIONALE

1.1 Plant sterols and stanols

Plant sterols, which are normal components in our daily diet, exert the same cellular functions in plants as cholesterol does in animals. The average intake of plant sterols in Western countries is approximately 250 mg/day, mainly derived from vegetable oils, cereals, nuts, seeds, fruits and vegetables. The most abundant plant sterols in the human diet are sitosterol, campesterol and stigmasterol. Plant stanols are the saturated derivatives of plant sterols. The major plant stanols are sitostanol and campestanol, which are much less abundant in nature than plant sterols are. Plant sterols and stanols are structurally related to cholesterol, but they have a different side-chain configuration. As humans are unable to synthesize plant sterols and stanols, their serum concentrations are mainly determined by intestinal absorption. Due to their low absorption, serum concentrations are less than 1% of that of serum cholesterol [1, 2].

Plant sterols and stanols lower cholesterol absorption in the intestine and therefore reduce serum LDL cholesterol concentrations [3]. At an intake of approximately 2.5 gram per day, functional foods enriched with phytosterols decrease LDL cholesterol up to 10% [4]. These products are therefore widely available and used to lower the risk for coronary heart disease via dietary means. They are generally recognized as safe (GRAS) [4, 5] and part of guidelines for lowering cardiovascular risk in several countries [6].

1.2 Oxyphytosterols

As already indicated, plant sterols are structurally related to cholesterol. Therefore, they can also be oxidized. The side-chain of cholesterol can be oxidized enzymatically and the nucleus non-enzymatically, which is probably a radical driven process. For plant sterols, only the nucleus can be oxidized. Due to sterical hindrance caused by the different side-chain structure of plant sterols as compared to cholesterol, the side-chain of plant sterols cannot be oxidized. The ring-oxidation products are potentially atherogenic, at least for cholesterol oxidation products.

As an increased intake of plant sterols elevates their serum concentrations [7], it is likely that serum oxyphytosterols concentrations also increase. Plant stanols do not have a double bond in the steroid nucleus and can therefore not be oxidized [8]. In addition, plant stanols lower not only the absorption of cholesterol, but also that of plant sterols. Thus, plant stanols

not only lower serum LDL cholesterol concentrations, but also those of serum plant sterols. Therefore it can be hypothesized that plant stanol consumption lowers oxyphytosterol concentrations. This is important as non-conclusive evidence from animal studies suggests that oxyphytosterols are – like oxidation products of cholesterol – atherogenic [8]. This justifies the evaluation of serum oxyphytosterol concentrations in humans consuming products enriched with plant sterols or stanols.

The concentrations of oxyphytosterols in human plasma have so far been measured in two cross-sectional studies. In a first study by Plat et al [9] oxyphytosterols could be identified in serum of sitosterolaemic patients, but not in serum from healthy volunteers. Later, Grandgirard used a quantification method with a lower limit of detection and was able to identify concentrations of oxyphytosterols in plasma from healthy volunteers [10]. In the mean time the methodology to analyze serum oxyphytosterol concentrations has much improved. Therefore it is now possible to analyze much lower concentrations as compared to a few years ago. Until now, no studies have examined serum concentrations of oxyphytosterols in healthy human subjects in response to an increased intake of plant sterols and stanols consumed as part of their daily diet for a few weeks.
As humans are in the postprandial state for most of the day [11], it is also important to monitor changes in oxyphytosterol concentrations after a meal containing plant sterols and stanols. A postprandial approach will also give the possibility to study postprandial changes in cholesterol and triacylglycerol metabolism as an increase in postprandial lipids is associated with an increased risk for coronary heart disease [12]. Studies by Agren et al [13] and Relas et al [14] have shown that it is possible to follow the postprandial chylomicron response in terms of cholesterol and triacylglycerol content.

1.3 Previous research

Until now, there is little information available on the presence of oxyphytosterols in healthy human subjects. The most important oxyphytosterols that have been identified are; 5α,6α-epoxy-sitosterol, 7=O-sitosterol, 7β-OH-sitosterol, en 3β,5α,6β-tri-hydroxy-sitosterol. The same oxyphytosterols can be identified for campesterol [9]. There hasn't been any research performed on the location where the oxyphytosterols are produced, on the breakdown route and speed or on the pathophysiological effect. Therefore information is still lacking on this topic. It is difficult to say anything about the expected concentration of oxyphytosterols, as there has been only one published study that measured concentrations in serum healthy human subjects. Based on an article in sitosterolemic subjects, we expect an increase of 17 ng/ml in oxyphytosterol concentration upon consumption of plant sterols.

As mentioned earlier, a study by Plat et al [9] showed that more oxyphytosterols are present in serum when the concentration plant sterols is higher. When you combine these results with the result of a study by Naumann et al [15], who showed that the increase in plant sterols is proportional to the plant sterol intake, you can expect a dose-dependant effect for oxyphytosterols as well.

2. OBJECTIVES

The ***major objective*** of the present study is to examine the effects of dietary plant sterols and stanols on fasting serum concentrations of oxyphytosterols. The ***minor objective*** is to investigate the effects of these products on changes in postprandial serum oxyphytosterol concentrations.

Major null hypothesis, H_0:

There is no association between the change in fasting serum plant sterol concentrations induced by consumption of phytosterol-enriched products (3.0 gram sterols or stanols / day for 4 weeks) and the change in fasting serum oxyphytosterol concentrations in healthy men and women.

Major alternative hypothesis, H_a:

There is an association between the change in fasting serum plant sterol concentrations induced by consumption of phytosterol-enriched products (3.0 gram sterols or stanols / day for 4 weeks) and the change in fasting serum oxyphytosterol concentrations in healthy men and women.

Minor null hypothesis, H_0:

There is no association between the change in postprandial serum plant sterol concentrations induced by consumption of a meal containing phytosterol-enriched products (3.0 gram sterols or stanols) and the change in postprandial serum concentrations of oxyphytosterols.

Minor alternative hypothesis, H_a:

There is an association between the change in postprandial serum plant sterol concentrations induced by consumption of a meal containing phytosterol-enriched products (3.0 gram sterols or stanols) and the change in postprandial serum concentrations of oxyphytosterols.

3. STUDY DESIGN

This study is a randomized, placebo-controlled, cross-over study with 48 men and women. Subjects will be recruited in and near the vicinity of Maastricht by means of posters distributed in university and hospital buildings, advertisements in local newspapers, the hospital bulletin and on the internet (www.digi-prik.nl). Subjects who have participated in earlier studies and indicated to be interested in participation in other studies will be contacted. Before screening, subjects will be informed about the procedures during the study via oral and written information (see information brochure). After information is given, subjects can consider participation for at least 1 day. When positive, informed consent will be obtained before start of the study. Participation will be on voluntary basis and people who are willing to participate will be invited for a screening visit. Subjects will be informed about their results obtained during the screening and advised to consult their general practitioner when values of the screening parameters are outside the normal ranges. When subjects fulfill all inclusion criteria, they may enter the study.

During the first 4 weeks of the study (period I), 16 subjects will be provided with control margarine, 16 subjects will consume a margarine enriched with plant sterol esters and 16 subjects will consume a margarine enriched with plant stanol esters. Exactly 20 gram of the margarine should be consumed daily, divided over at least two eating moments. Subjects do not add the margarines to their daily diet, but are advised to replace their normal margarine / butter for the margarines provided by us. Therefore it is not expected that people will change in weight, as was also the case in our earlier trials with these margarines. After period I, the subjects will return to their normal eating habits for 4 weeks (wash-out period). Then, the subjects will cross-over to another dietary regime in period II. Hereafter they will again return to their normal eating habits for 4 weeks and cross-over to the last dietary regime in period III. There will be no dietary restriction as long as dietary habits remain stable over the entire study. During the wash-out period, the subjects are not allowed to consume plant sterol- and stanol enriched products.

All subjects must adhered to each dietary regime, meaning consumption of control margarine, plant sterol-enriched margarine and plant stanol-enriched margarine during the three periods. Compliance can be monitored by measurement of serum plant sterol and stanol concentrations. As depicted in table 1 (appendix 1), the subjects will come to the department at the beginning of period I, II and III (weeks 1, 9, and 17). During these visits, a fasting blood sample will be taken and body weight is measured. These blood samples are used to evaluate whether the wash out periods have been long enough since plant sterol and stanol concentrations in weeks 9 and 17 have to be similar to baseline levels (week 1). Based on earlier studies we expect 4 weeks to be long enough but it is necessary to confirm this in this study. At the end of all three periods, subjects will also come to the department

for a fasting blood sample (weeks 3, 11 and 19) and to participate in a postprandial test (weeks 4, 12, 20), which will be explained later into more detail.

All subjects will pick up their supply of products when they visit the department for the blood sampling. The margarines will be provided in one-week portions of 140 gram. Cups that are left over at the end of the week must be returned to the department and will be weighed. The margarine tubs will be color-coded to blind both the subjects and the investigators.

The subjects will record in a diary the amounts and times of consumption of the margarines and they will also be asked to record every signs of illnesses, medication used and any deviations from protocol. Furthermore, they will be urged not to change their dietary habits, level of physical activity, use of alcohol or of oral contraceptives throughout the study.

A registered dietician will check these dairies at each visit that will be combined with picking up a new supply of the products. At the end of the three periods I, II and III, the subjects will also fill out a food frequency questionnaire to estimate their habitual intake over the previous 4 week period (weeks 4, 12, 20).

4. STUDY POPULATION

4.1 Population

Subjects will include 48 apparently healthy volunteers, aged between 18 and 70 years that have to fulfill the inclusion criteria as described below. From our previous experiences, we know that a high proportion of people of >70 years do have one or more of the exclusion criteria as formulated in paragraph 4.4. Because we do not want to encumber these subjects unnecessarily, we have decided to set the upper limit for age at 70 years.

4.2 Screening visit

The subjects will be invited for a screening visit, this visit will include recording of:

- body weight
- length
- blood pressure (diastolic and systolic)
- blood parameters (serum total cholesterol, triacylglycerol and glucose concentrations)
- use of medication
- current disease and history of coronary heart disease

Subjects will come to the department twice for two screening visits. During the first screening visit, the subjects will be weighed, blood pressure will determined in four-fold (the first

measurement will be discarded and the last three measurement will be averaged), body height will determined and a venous blood sample (1*3.5 ml and 1*2 ml) will be drawn for analysis of serum total cholesterol, HDL cholesterol, triacylglycerol and glucose concentrations.

During the second screening visit, body weight and blood pressure will be determined again and a second venous blood sample (1*3.5 ml and 1*2 ml) will be drawn for analysis of serum total cholesterol, HDL cholesterol, triacylglycerol and glucose concentrations.

In case the subjects are a blood donor, they will be told that they cannot donate blood for 4 weeks after completion of the study.

4.3 Inclusion criteria

The inclusion criteria are:

- aged between 18 and 70 years
- Body Mass Index (BMI) between 20-30 kg/m^2
- mean serum total cholesterol < 7.8 mmol/L
- mean serum triacylglycerol < 3.0 mmol/L
- mean plasma glucose < 6.1 mmol/L

4.4 Exclusion criteria

The exclusion criteria are:

- unstable body weight (weight gain or loss > 3 kg in the past two months)
- active cardiovascular diseases like congestive heart failure or recent (<6 months) event (acute myocardial infarction, cerebral vascular incident)
- severe medical conditions that might interfere with the study such as epilepsy, asthma, chronic obstructive pulmonary disease, inflammatory bowel disease and rheumatoid arthritis)
- indication for treatment with cholesterol-lowering drugs according to the Dutch Cholesterol Consensus
- use of medication such as corticosteroids, diuretics or lipid lowering therapy
- abuse of drug or alcohol (>21 units per week)
- not willing to stop the consumption of vitamin supplements, fish oil capsules or products rich in sterol or stanol esters 4 weeks before the start of the study (wash-in period)
- use of an investigational product within another biomedical study within the previous

month
- pregnant or breast-feeding women
- not willing to give up being a blood donor (or having donated blood) from 8 weeks before the start of the study and during the study
- current smoker
- anemia. with a Hb-level below 7.5 mmol/L for men and below 7.0 mmol/L for women, as indicated by the blood bank of Maastricht

4.5 Sample size calculation

Using the primary outcome parameter, i.e a true change of 17 ng/mL in serum oxyphytosterol concentrations and the known within-subject variation on the response of 35 ng/mL, it can be calculated that we need 44 subjects to have a power of 80% to detect the indicated difference, if we consider a P-value < 0.017 (to account for multiple comparisons between groups) to be statistically significant. The expected drop-out rate is 10%. Therefore, 48 volunteers will be recruited.

5. TREATMENT OF SUBJECTS

5.1 Placebo controlled dietary intervention

Subjects will be asked to consume 20 gram control margarine or margarine with plant sterol esters or margarine with plant stanol esters for three periods of four weeks. They will be asked to consume this amount of margarine daily, divided over two eating moments. Each test-period is separated by a wash-out period of 4 weeks, during which the subjects will return to their normal eating habits, however they are not allowed to consume plant sterol- and stanol enriched products. They will visit the department at weeks 1, 9 and 17 to give a blood sample. At weeks 3, 11 and 19 they will visit the department to pick-up a new supply of margarines, to undergo a blood sampling and to talk to the dietician. At weeks 4, 12 and 20 they will undergo a postprandial test, which is explained into more detail below (§ 5.2).

5.2 Postprandial test

After a 12-hour fast (from 20.00 the proceeding day), subjects will come to the department at 08.00 by public transport or car to standardize measurements as much as possible. After arrival, the subjects will be weighed and will rest for 15 minutes before an intravenous cannula is inserted into a vein. At T=0 min (T0), a blood sample is collected. Subjects are then requested to consume a test breakfast (see below) within 10 minutes. Subsequent

blood samples are collected at T=15 min, T=30 min, T=45 min, T=60 min, T=90 min, T=120 min, T=180 and T=240. After the 4-hour sample, subjects will consume a second meal (lunch) in order to further follow uptake of dietary cholesterol and plant sterols / stanols (second meal effect). Further blood samples are drawn at T=300, T=360, T=420 en T=480. The subjects are allowed to drink water and are free to walk around during the entire 8 hours period.

5.3 Composition of the experimental products and menus

The margarines are based on soysterols esterified with rapeseed oil fatty acids and the plant stanol esters are also esterified with rapeseed oil fatty acids. All margarines contain 40% absorbable fats ("light margarine"). The control margarine does not contain any plant sterol or stanol esters, while 20 gram of the experimental margarine will provide 3.0 gram of either plant sterols or stanols as its fatty acid esters. The composition of the products will be comparable to the products currently available in Dutch and Belgian supermarkets.

The margarines will be similar in colour, taste, absorbable fat content and caloric value. Raisio Nutrition Ltd will provide all the margarines (appendix 2: composition and appendix 3: safety statement). Products will be manufactured according to HACCP-principles (appendix 4: HACCP analysis). Raisio will take care of the costs for the production, packaging and transport of the margarines and for quality control. Products will be stored at the department of Human Biology at -20°C. The margarines will be produced in 1 batch after which Raisio will check the composition of the margarines and the specific content of plant sterols and stanols. Hereafter, the margarines will be send to our department and we will obtain the exact analysis of the batch which will be sent to the METC.

The meals (breakfast and lunch) for the postprandial test will consist of a standardised milk shake (T=0) and the amount of macronutrients and cholesterol will be the same for each subject. The three breakfast and lunch menus (weeks 4, 12, 20) will be iso-energetic. The only component that will differ between the three menus is the amount of plant sterol or stanol esters.

The nutritional composition of the milkshakes is shown in table 2 and this composition will be similar compared to the composition of milkshakes that were used in earlier trials at our department. All of the ingredients of the milkshake are available in the supermarket for consumption. These products are bought by our dietician and stored as indicated on the labels in our diet kitchen (room 3.247 uns50) in the refrigerator or on room temperature. The plant sterols and stanols that are used in the milkshake will be derived from the plant sterol- or stanol enriched margarines. The shakes will be prepared fresh on the morning of the test days in our diet kitchen and stored there at 4°C till consumption. As can be seen in table 2, the fat content of the milkshake is 50 gram. The amount of 50 gram of fat has been chosen,

as it represents a typical fat load in the western diet as supplied by a dinner, producing the necessary degree of lipaemia [16].

Table 2. Nutritional composition of the breakfast and lunch shake

	**Breakfast shake**		**Lunch shake**	
**Nutrient**	**Amount**	**En% daily intake**	**Amount**	**En% daily intake**
**Energy**	731 kcal	32	731 kcal	32
**Fat**	49.8 g	19.9	49.8 g	19.9
**SAFA**	20.1 g	8.0	20.1 g	8.0
**MUFA**	18.4 g	7.4	184.4 g	7.4
**PUFA**	7.6 g	3.0	7.6 g	3.0
**Protein**	11.5 g	2	11.5 g	2
**Carbohydrate**	59.2 g	10.5	59.2 g	10.5
**Cholesterol**	250.2 mg	-	-	-
**Plant sterols / stanols**	0 g (control) or 3.0 g (experimental)	-	-	-
**Retinyl palmitate**	100.000 IU	-	-	-

SAFA: saturated fatty acids, MUFA: monounsaturated fatty acids, PUFA: polyunsaturated fatty acids

The volunteers will receive a second milkshake after 4 hours. The composition will be the same as the first milkshake, but without the added plant sterols/stanols or cholesterol (table 2). This second meal will induce a further uptake of the plant sterols/stanols and cholesterol that were ingested during the first meal [17, 18]. As we are interested in the uptake of phytosterols, we are aiming for the most efficient uptake and this will most likely be achieved after the second meal. This phenomenon is referred to as second meal effect, which means that cholesterol ingested during the first meal, will appear in the circulation when a second meal has been consumed. In other words there is a delay in the appearance of consumed sterols which can be monitored by prolonged follow up (4 - 8 h) after the first meal while supplying a second meal halfway.

The first shake contains retinyl palmitate, to follow the intestinal handling of dietary triacylglycerol during the rest of the day. The first milkshake (T=0) will therefore be enriched with 100.000 IU for an person of average body weight [19]. Retinyl palmitate is an esterified form of vitamin A. It has been commonly used in research to label intestinally-derived chylomicron particles. The presence and concentration of retinyl esters in the plasma can be used as an indicator of dietary triacylglycerol uptake into chylomicrons and chylomicron remnants [20]. Retinyl palmitate is safe for human consumption (see appendix 5). The LD50 (toxicology-index) is >4000 mg/kg body weight and the amount that we will administer is 1.25 mg/kg body weight. As a comparison, the LD50 of normal kitchen salt (NaCl) is also >4000 mg/kg body weight [21].

The retinyl palmitate will be replaced before its expiration date and a new analysis certificate will be obtained and sent to the METC.

6. METHODS

6.1 Randomization, blinding and treatment allocation

The subjects will receive the three experimental periods in a randomized order, based upon a computer-generated table with random numbers. For this, a categorical list in logical order will be created including all interventions per subject. After addition of a computer-generated list of random numbers, the list will be sorted by subject number and random number, resulting in a randomized list of treatment allocation. The randomization code will be broken after statistical analyses are completed. To blind the researchers and the subjects, the margarine cups will be coded. For randomization, subjects will be stratified for age, gender and BMI.

6.2 Study procedures

Subjects will be asked to consume 20 gram control margarine or margarine with plant sterol esters or margarine with plant stanol esters for three periods of four weeks. Each test-period is separated by a wash-out period of 4 weeks, where the subjects will return to their normal eating habits. They will visit the department in week 1, 9 and 17 to give a blood sample. In week 3, 11 and 19 they will visit the department to pick-up a new supply of margarine packages, to undergo a blood sampling and to talk to the dietician. In week 4, 12 and 20 they will undergo a postprandial test, therefore subjects will come to the department at 08.00 by public transport or car to standardize measurements as much as possible after a 12-hour fast (from 20.00 the proceeding day). After arrival, the subjects will be weighed and rest for 15 minutes before an intravenous cannula is inserted into a vein. At T=0 min (T0), a blood

sample is collected. Subjects are then requested to consume a test breakfast (see below) within 10 minutes. Subsequent blood samples are collected at T=15 min, T=30 min, T=45 min, T=60 min, T=90 min, T=120 min, T=180 and T=240. After the 4-hour sample, subjects will consume a second meal (lunch) in order to further follow uptake of dietary cholesterol and plant sterols / stanols (second meal effect). Further blood samples are drawn at T=300, T=360, T=420 en T=480. The subjects are allowed to drink water and are free to walk around during the entire 8 hours period.

The amount of blood sampled at each occasion is given in table 4.

Table 4. Blood sampling scheme

**Week**	**1**	**3**	**4**	**9**	**11**	**12**	**17**	**19**	**20**
Action	1 blood sample	1 blood sample	Postprandial test	1 blood sample	1 blood sample	Postprandial test	1 blood sample	1 blood sample	Postprandial test
Blood (ml)	3.5	3.5	164.5	3.5	3.5	164.5	3.5	3.5	164.5

The total amount of blood drawn will be 514.5 ml per person during the whole study. As suggested by the METC in a previous study proposal, some precaution measures will be taken. For this reason, subjects are not allowed to have donated blood 8 weeks prior to participation. In addition, Hb-levels will be checked immediately at each test day at T=0. A normal Hb-level for a man is > 7.5 mmol/L and for a women > 7.0 mmol/L as indicated by the blood bank of Maastricht. If Hb concentrations are below 7.5 mmol/L (men) or 7.0 mmol/L (women), the test will be stopped.

Although 514.5 ml seems a lot, it should be realized that this is divided over a period of 20 weeks divided over 9 sampling moments with 4-week intervals. The maximum amount sampled at once is 164.5 ml.

Blood samples drawn in week 1, 9 and 17 will be used to check whether the wash-out period has been successful. Samples drawn in week 3, 11 and 19 are needed to obtain a reliable determination of serum lipid concentrations, as these tend to fluctuate over time. In week 4, 12 and 20, the samples drawn at T=0 will be used to determine fasting blood concentrations. The postprandial test will take place consecutively to determine postprandial concentrations of all study parameters. The time points upon which the blood samples are drawn are chosen to establish a time-response curve for plant sterol and stanol concentrations as well as for oxyphytosterol concentrations. Next to this, the timepoints are indicative for the plasma TAG response during the entire postprandial period. Blood samples are drawn every

15 minutes during the first hour to establish a glucose response curve. In appendix 5, tables 6 and 7 list the amounts of blood needed to perform the chosen analysis for this study.

6.3 Study parameters

We will analyze parameters related to the research questions as described below. Based on new insights related to the original research question, it may be decided to analyze the samples for other parameters. Therefore, subjects will explicitly be asked in the informed consent form to consent (or not) with this approach.

6.3.1 Metabolic risk markers

Fasting and Postprandial samples

Plant sterol and stanol metabolism:

- Serum plant sterol and stanol concentrations and serum oxysterol and oxyphytosterol concentrations will be determined during the entire postprandial curves in weeks 4, 12 and 20 at 0, 15, 30, 45, 60, 90, 120, and at hourly intervals up to 8 h and will provide information on the plant sterol and stanol metabolism and the level of oxidation during the postprandial phase. Plant sterol and stanol concentrations will also be measured at time point in week 1, week 3, week 4 (time 0), week 9, week 11, week 12 (time 0), weeks 17, week 19 and week 20 (time 0), to provide information on the fasting concentrations. Butylated hydroxy toluene (BHT) will be added to the EDTA blood collection tubes to prevent auto-oxidation of the sterols and other components in the blood.

Chylomicron isolation

The chylomicron fraction will be isolated from EDTA plasma, by means of ultracentrifugation at time point 0, 2, 4, 6 and 8 h.

- Retinol concentrations will be determined in this fraction to assess the absorption of retinyl palmitate and to provide information on the TAG absorption and the chylomicron particle composition.
- The cholesterol and triacylglycerol content will be measured to provide information on the absorption and incorporation in the chylomicron particle of respectively dietary cholesterol and TAG.

Postprandial lipemia:

- Serum total and HDL cholesterol will be measured at time point in week 1, week 3, week 4 (time 0), week 9, week 11, week 12 (time 0), week 17, week 19 and week 20 (time 0), to provide information on the fasting cholesterol concentrations
- Serum TAG will be measured during the entire postprandial time curves in weeks 4, 12 and 20 at 0, 15, 30, 45, 60, 90, 120, and at hourly intervals up to 8 h, and be used to determine the overall level of postprandial lipemia.
- Plasma free fatty acids will be measured during the entire postprandial curves in weeks 4, 12 and 20 at 0, 15, 30, 45, 60, 90, 120, and at hourly intervals up to 8 h, to provide additional information on the postprandial lipemic response.
- Apolipoprotein B48 (apoB48) concentration will be measured during the entire postprandial curves in weeks 4, 12 and 20 at 0, 15, 30, 45, 60, 90, 120, and at hourly intervals up to 8 h, and be used alongside measurement of serum TAG concentrations to provide information on the rates of TAG absorption and chylomicron particle size.
- ApoB100 concentration will be measured at time point in week 1, week 3, week 4 (time 0), week 9, week 11, week 12 (time 0), week 17, week 19 and week 20 (time 0), to provide information on the fasting concentrations.
- ApoA-1 concentration will be measured at time point in week 1, week 3, week 4 (time 0), week 9, week 11, week 12 (time 0), week 17, week 19 and week 20 (time 0), to provide information on the fasting concentrations.

Postprandial glycemia and insulinemia:

- Plasma glucose concentrations will be measured during the entire postprandial curves in weeks 4, 12 and 20 at 0, 15, 30, 45, 60, 90, 120, and at hourly intervals up to 8 h, to determine postprandial glycemic response.
- Insulin concentration will be measured during the entire postprandial curves in weeks 4, 12 and 20 at 0, 15, 30, 45, 60, 90, 120, and at hourly intervals up to 8 h, to assess insulin metabolism.

Low-grade inflammation and endothelial dysfunction:

- hsCRP, TNF-α, TNF-RI, TNFR-II, IL-6, MCP-1, vWF, sICAM-1, sVCAM-1 and sE-selectin will be measured at time point in week 4 (time 0), week 12 (time 0) and week 20 (time 0), to assess fasting concentrations of low-grade inflammation and endothelial dysfunction.

6.3.2 Food intake

Habitual food intake will be measured at the end of each test period using a validated food frequency questionnaire, which will be checked in the presence of a dietician immediately.

6.3.3 Body weight

Body weight without shoes and heavy clothing will be measured at the beginning and at the end of each test period.

6.4 Withdrawal of individual subjects

Subjects can leave the study at any time for any reason if they wish to do so without any consequences. The investigator can decide to withdraw a subject from the study for urgent medical reasons or non-compliance.

6.5 Replacement of individual subjects after withdrawal

After withdrawal, subjects will not be replaced.

6.6 Follow-up of subjects withdrawn from treatment

After withdrawal, no follow-up of subjects will take place. In case of withdrawal due to medical compliance, subjects will be referred to a general practitioner.

7. SAFETY REPORTING

7.1 Section 10 WMO event

In accordance to section 10, subsection 1, of the WMO, the investigator will inform the subjects and the reviewing accredited METC if anything occurs, on the basis of which it appears that the disadvantages of participation may be significantly greater than was foreseen in the research proposal. The study will be suspended pending further review by the accredited METC, except insofar as suspension would jeopardize the subjects' health. The investigator will take care that all subjects are kept informed.

7.2 Adverse and serious adverse events

Adverse events are defined as any undesirable experience occurring to a subject during a clinical trial, whether or not considered related to the investigational drug. All adverse events

reported spontaneously by the subject or observed by the investigator or his staff will be recorded.

A serious adverse event (SAE) is any untoward medical occurrence or effect that at any dose results in death:

- is life threatening (at the time of the event)
- requires hospitalization or prolongation of existing inpatients' hospitalization
- results in persistent or significant disability or incapacity
- is a congenital anomaly or birth defect
- is a new event of the trial likely to affect the safety of the subjects, such as an unexpected outcome of an adverse reaction, lack of efficacy of an IMP used for the treatment of a life threatening disease, major safety finding from a newly completed animal study, etc.

All SAEs will be reported to the accredited METC that approved the protocol, according to the requirements of that METC.

7.3 Follow-up of adverse events

All adverse events will be followed until they have abated, or until a stable situation has been reached. Depending on the event, follow up may require additional tests or medical procedures as indicated, and/or referral to the general physician.

8. STATISTICAL ANALYSIS

Differences between the experimental groups and the control group will be calculated and effects of the intervention will be examined by analysis of variance (ANOVA) test. A *P*-value <0.017 is considered to be statistically significant.

9. ETHICAL CONSIDERATIONS

9.1 Regulation statement

The study has to be approved by the Medical Ethical Committee of the University of Maastricht.

9.2 Recruitment and consent

Subjects will be recruited among men and women in and near the vicinity of Maastricht by means of posters distributed in university and hospital buildings, advertisements in local

newspapers, the hospital bulletin and on the internet (www.digi-prik.nl). In addition, subjects who have participated in earlier studies and who have indicated that they are interested in other studies, will be sent an information brochure. Before the start of the study volunteers will be given oral and written information about the aim of the study. After information is given, subjects can consider participation for at least 1 day. Hereafter, informed consent will be obtained before start of the study. Participation will be on voluntary basis and people who are willing to participate will be invited for a screening visit.

Subjects are free to approach the independent physician for further information and questions, and to withdraw at any stage of the study without further explanation.

9.3 Privacy

Before screening, subjects are informed about the procedures and informed consent will be obtained. Personal data will be handled confidentially and be stored in a password-protected file, to which only the investigators have access. Samples will be coded and destroyed after 5 years. Only the investigators have access to the code. Subjects can leave the study at any time for any reason if they wish to do so without any consequences.

9.4 Benefits and risks assessment, group relatedness

The subjects will record in diaries any signs of illness, medication used, any deviations from the protocol, and any experienced side effects (headache, stomach complaints, nausea, bloated feeling, flatulence, diarrhea, constipation, itching, eruptions/rashes, fatigue, and dizziness).

The experimental products are safe (appendix 3) and manufactured according to generally accepted procedures and Good Manufacturing Practices. Venipunctures can occasionally cause a local haematoma or a bruise. Some participants may report pain during venipuncture. There is no direct benefit for the participants.

9.5 Compensation for injury

The university of Maastricht has insurance, which is in accordance with the legal requirements in the Netherlands (Article 7 WMO and the Measure regarding Compulsory Insurance for Clinical Research in Humans of 23rd June 2003). This insurance provides cover for damage to research subjects through injury or death caused by the study.

1. € 450.000 (i.e. four hundred and fifty thousand Euro) for death or injury for each subject, who participates in the Research;

2. € 3.500.000 (i.e. three million five hundred thousand Euro) for death or injury for all subjects, who participate in the Research;
3. € 5.000.000 (i.e. five million Euro) for the total damage incurred by the organization for all damage disclosed by scientific research for the Sponsor as 'verrichter' in the meaning of said Act in each year of insurance coverage.

The insurance applies to the damage that becomes apparent during the study or within 4 years after the end of the study. There is also liability insurance.

9.6 Incentives

The time invested by subjects is approximately 27 hours, as can be seen in table 5. During the postprandial tests, subjects are free to perform paper work or to study, except for the moments of blood sampling. Compensation for participation will be €225. This amount is based on the fact that subjects will undergo a postprandial test 3 times (3*€50) and will have to come another 6 times to the department for blood sampling and picking up margarine supplies (€75), which sums up tot a total of €225. After withdrawal, the compensation will be pro rata. There will be no financial reward for the screening visits, as the time invested is minimal, while subjects will receive a free breakfast and a free health check. Traveling costs will be compensated (public transport or €0.19/km for car travel). The test products will be provided for free.

Table 5. Time investment of participants

Week	Blood sampling	Picking up supply and talk with dietician	Food frequency questionnaire	Postprandial test	Total
**Screening**					
-1	20 min				20 min
-2	20 min				20 min
**Intervention period**					
1	10 min	5 min			15 min
3	10 min	5 min			15 min
4			20 min	480 min	500 min
9	10 min	5 min			15 min
11	10 min	5 min			15 min
12			20 min	480 min	500 min
17	10 min	5 min			15 min
19	10 min	5 min			15 min
20			20 min	480 min	500 min
**Total time invested**					**27 hours**

10. ADMINISTRATIVE ASPECTS AND PUBLICATION

At the start of the study, subjects will be assigned a random number that will not change during the study. This number is linked with the name, address, date of birth, and telephone number of the subject in a password-protected file. Only members of the project team can access this file. For all other purposes, the random number will be used for subject identification.

Amendments are changes made to the research after a favorable opinion by the accredited METC has been given. All amendments will be notified to the METC that gave a favorable opinion.

The investigator will notify the accredited METC of the end of the study within a period of 90 days. The end of the study is defined as the last patient's last visit. In case the study is ended prematurely, the investigator will notify the accredited METC, including the reasons for the premature termination.

Publication policy is in agreement with the CCMO publication statement. The results of the

study will be published in peer-reviewed scientific journals. Both positive and negative results of the study will be disclosed. The principal investigator will always try to publish and/or present results to the general public.

11. REFERENCES

[1] Brufau G, Canela MA, Rafecas M. Phytosterols: physiologic and metabolic aspects related to cholesterol-lowering properties. Nutr Res. 2008 Apr;28(4):217-25.
[2] Gylling H, Miettinen TA. The effect of plant stanol- and sterol-enriched foods on lipid metabolism, serum lipids and coronary heart disease. Ann Clin Biochem. 2005 Jul;42(Pt 4):254-63.
[3] Abumweis SS, Barake R, Jones PJ. Plant sterols/stanols as cholesterol lowering agents: A meta-analysis of randomized controlled trials. Food Nutr Res. 2008;52.
[4] Katan MB, Grundy SM, Jones P, Law M, Miettinen T, Paoletti R. Efficacy and safety of plant stanols and sterols in the management of blood cholesterol levels. Mayo Clin Proc. 2003 Aug;78(8):965-78.
[5] Plat J, Mensink RP. Plant stanol and sterol esters in the control of blood cholesterol levels: mechanism and safety aspects. Am J Cardiol. 2005 Jul 4;96(1A):15D-22D.
[6] Executive Summary of The Third Report of The National Cholesterol Education Program (NCEP) Expert Panel on Detection, Evaluation, And Treatment of High Blood Cholesterol In Adults (Adult Treatment Panel III). Jama. 2001 May 16;285(19):2486-97.
[7] Hallikainen MA, Sarkkinen ES, Gylling H, Erkkila AT, Uusitupa MI. Comparison of the effects of plant sterol ester and plant stanol ester-enriched margarines in lowering serum cholesterol concentrations in hypercholesterolaemic subjects on a low-fat diet. Eur J Clin Nutr. 2000 Sep;54(9):715-25.
[8] Hovenkamp E, Demonty I, Plat J, Lutjohann D, Mensink RP, Trautwein EA. Biological effects of oxidized phytosterols: a review of the current knowledge. Prog Lipid Res. 2008 Jan;47(1):37-49.
[9] Plat J, Brzezinka H, Lutjohann D, Mensink RP, von Bergmann K. Oxidized plant sterols in human serum and lipid infusions as measured by combined gas-liquid chromatography-mass spectrometry. J Lipid Res. 2001 Dec;42(12):2030-8.
[10] Grandgirard A, Martine L, Demaison L, Cordelet C, Joffre C, Berdeaux O, et al. Oxyphytosterols are present in plasma of healthy human subjects. Br J Nutr. 2004 Jan;91(1):101-6.
[11] Lairon D. Macronutrient intake and modulation on chylomicron production and clearance. Atheroscler Suppl. 2008 Sep;9(2):45-8.
[12] Patsch JR, Miesenbock G, Hopferwieser T, Muhlberger V, Knapp E, Dunn JK, et al. Relation of triglyceride metabolism and coronary artery disease. Studies in the postprandial state. Arterioscler Thromb. 1992 Nov;12(11):1336-45.
[13] Agren JJ, Hallikainen M, Vidgren H, Miettinen TA, Gylling H. Postprandial lipemic response and lipoprotein composition in subjects with low or high cholesterol absorption efficiency. Clin Chim Acta. 2006 Apr;366(1-2):309-15.
[14] Relas H, Gylling H, Miettinen TA. Effect of stanol ester on postabsorptive squalene and retinyl palmitate. Metabolism. 2000 Apr;49(4):473-8.
[15] Naumann E, Plat J, Mensink RP. Changes in serum concentrations of noncholesterol sterols and lipoproteins in healthy subjects do not depend on the ratio of plant sterols to stanols in the diet. J Nutr. 2003 Sep;133(9):2741-7.
[16] Lopez-Miranda J, Williams C, Lairon D. Dietary, physiological, genetic and pathological influences on postprandial lipid metabolism. Br J Nutr. 2007 Sep;98(3):458-73.
[17] Evans K, Kuusela PJ, Cruz ML, Wilhelmova I, Fielding BA, Frayn KN. Rapid chylomicron appearance following sequential meals: effects of second meal composition. Br J Nutr. 1998 May;79(5):425-9.

[18] Silva KD, Wright JW, Williams CM, Lovegrove JA. Meal ingestion provokes entry of lipoproteins containing fat from the previous meal: possible metabolic implications. Eur J Nutr. 2005 Sep;44(6):377-83.
[19] Cabezas MC, de Bruin TW, Jansen H, Kock LA, Kortlandt W, Erkelens DW. Impaired chylomicron remnant clearance in familial combined hyperlipidemia. Arterioscler Thromb. 1993 Jun;13(6):804-14.
[20] Silva KD, Williams CM, Lovegrove JA. Use of water-miscible retinyl palmitate as markers of chylomicrons gives earlier peak response of plasma retinyl esters compared with oil-soluble retinyl palmitate. Br J Nutr. 2001 Oct;86(4):427-32.
[21] Bollinger TK, Mineau P, Wickstrom ML. Toxicity of sodium chloride to house sparrows (Passer domesticus). J Wildl Dis. 2005 Apr;41(2):363-70.
