# Supplementary material for: Effects of a Plant Sterol or Stanol Enriched Mixed Meal on Postprandial Lipid Metabolism in Healthy Subjects
Source: PLoS One. 2016 Sep 9;11(9):e0160396. doi: 10.1371/journal.pone.0160396 (PMC5017646; doi:10.1371/journal.pone.0160396)
Supplement: S1 Table — (DOCX) [file pone.0160396.s004.docx]

Effects of a plant sterol or stanol enriched mixed meal on postprandial lipid metabolism in healthy subjects

Sabine Baumgartner^1^*, Ronald P. Mensink^1^ and Jogchum Plat^1^

^1^ Department of Human Biology, NUTRIM School of Nutrition and Translational Research in Metabolism, Maastricht University Medical Center, Maastricht, the Netherlands

* Corresponding author

E-mail: sabine.baumgartner@maastrichtuniversity.nl

**S1 Table. Baseline characteristics of subjects separated per age category**

|  | Age category I  18-35y (n=17) | Age category II  36-52y (n=11) | Age category III  53-69y (n=14) |
| --- | --- | --- | --- |
| Age (y) | 23 ± 4 | 46 ± 4 | 62 ± 4 |
| Male / female (n) | 5 / 12 | 4 / 7 | 8 / 6 |
| Weight (kg) | 73.0 ± 11.4 | 68.5 ± 12.6 | 78.2 ± 8.8 |
| Height (m) | 1.74 ± 0.10 | 1.69 ± 0.11 | 1.73 ± 0.06 |
| BMI (kg/m^2^) | 24.1 ± 2.3 | 23.9 ± 3.1 | 26.1 ± 2.7 |
| Glucose (mmol/L) | 4.82 ± 0.23 | 5.42 ± 0.36^a^ | 5.66 ± 0.58^a^ |
| HOMA-index^d^ | 1.71 ± 0.56 | 1.00 ± 0.28 | 1.40 ± 0.77 |
| TCH (mmol/L) | 5.09 ± 0.95 | 5.62 ± 1.15 | 6.60 ± 0.74^a,c^ |
| HDL-C (mmol/L) | 1.64 ± 0.36 | 1.81 ± 0.39 | 1.66 ± 0.39 |
| TC/HDL ratio^d^ | 3.22 ± 0.90 | 3.16 ± 0.70 | 4.19 ± 1.11^b,c^ |
| TG (mmol/L)^d^ | 1.15 ± 0.50 | 1.13 ± 0.49 | 1.09 ± 0.41 |

Data are means ± SD

Significant difference compared with age category I ^a^(*P* < 0.001), ^b^(*P* < 0.05)

Significant difference compared with age category II ^c^(P < 0.05)

Parameters were tested by a Kruskal-Wallis test for not normally distributed data^d^
