# Supplementary material for: Effects of a Plant Sterol or Stanol Enriched Mixed Meal on Postprandial Lipid Metabolism in Healthy Subjects
Source: PLoS One. 2016 Sep 9;11(9):e0160396. doi: 10.1371/journal.pone.0160396 (PMC5017646; doi:10.1371/journal.pone.0160396)
Supplement: S2 Table — (DOCX) [file pone.0160396.s005.docx]

Effects of a plant sterol or stanol enriched mixed meal on postprandial lipid metabolism in healthy subjects

Sabine Baumgartner^1^*, Ronald P. Mensink^1^ and Jogchum Plat^1^

^1^ Department of Human Biology, NUTRIM School of Nutrition and Translational Research in Metabolism, Maastricht University Medical Center, Maastricht, the Netherlands

* Corresponding author

E-mail: sabine.baumgartner@maastrichtuniversity.nl

**S2 Table. Fasting concentrations, iAUC and maximal increases from baseline in TG concentrations after consumption of a mixed meal containing no, or 3.0 gram of plant sterols or plant stanols separated per age category.**

|  | Control period | | | Sterol period | | | Stanol period | | |
| --- | --- | --- | --- | --- | --- | --- | --- | --- | --- |
|  | 18-35 y | 36-52 y | 53-69 y | 18-35 y | 36-52 y | 53-69 y | 18-35 y | 36-52 y | 53-69 y |
| Fasting (mmol/L) | 1.12 ± 0.47 | 1.16 ± 0.57 | 1.14 ± 0.41 | 1.11 ± 0.40 | 1.03 ± 0.53 | 1.17 ± 0.41 | 1.05 ± 0.39 | 0.88 ± 0.35 | 1.17 ± 0.49 |
| iAUC^T^ (mmol/L/min) | 181.6 ± 90.2 | 226.5 ± 120.0 | 325.5 ± 171.8 | 186.6 ± 135.9 | 202.9 ± 116.7 | 387.1 ± 212.6 | 163.4 ± 80.3 | 308.5 ± 191.2 | 405.1 ± 211.2 |
| iAUC^1^ (mmol/L/min) | 60.9 ± 28.2 | 68.4 ± 32.5 | 85.5 ± 53.3 | 64.8 ± 44.8 | 62.9 ± 39.2 | 110.0 ± 57.2 | 55.1 ± 25.3 | 81.1 ± 62.1 | 100.9 ± 48.1 |
| iAUC^2^ (mmol/L/min) | 58.9 ± 54.8 | 67.3 ± 60.3 | 102.4 ± 49.3 | 61.3 ± 44.0 | 45.7 ± 43.6 | 82.5 ± 55.5 | 48.9 ± 34.5 | 92.6 ± 69.5^a^ | 145.6 ± 72.2^b,c^ |
| MaxTG (mmol/L) | 0.93 ± 0.38 | 1.19 ± 0.55 | 1.62 ± 0.71 | 1.08 ± 0.54 | 1.13 ± 0.61 | 1.71 ± 0.72 | 0.97 ± 0.44 | 1.74 ± 0.98^a^ | 1.85 ± 0.92 |

Data are means ± SD

^a^ Trend for difference stanol period compared with sterol period (*P* = 0.07); ^b^ significant difference stanol period compared with sterol period (*P* < 0.01); ^c^ significant difference stanol period compared with control period (*P* < 0.05). Fasting concentration and iAUC^2^ in age category I were tested by Friedman’s test for not normally distributed data. Greenhouse-Geisser correction was applied for MaxTG concentrations in age category I. iAUC^T^: incremental AUC of the total TG response, iAUC^1^: incremental AUC after the 1^st^ meal (0-4h), iAUC^2^: incremental AUC after the 2^nd^ meal (4-8h).
