# Supplementary material for: Effects of a Plant Sterol or Stanol Enriched Mixed Meal on Postprandial Lipid Metabolism in Healthy Subjects
Source: PLoS One. 2016 Sep 9;11(9):e0160396. doi: 10.1371/journal.pone.0160396 (PMC5017646; doi:10.1371/journal.pone.0160396)
Supplement: S3 Table — (DOCX) [file pone.0160396.s006.docx]

Effects of a plant sterol or stanol enriched mixed meal on postprandial lipid metabolism in healthy subjects

Sabine Baumgartner^1^*, Ronald P. Mensink^1^ and Jogchum Plat^1^

^1^ Department of Human Biology, NUTRIM School of Nutrition and Translational Research in Metabolism, Maastricht University Medical Center, Maastricht, the Netherlands

* Corresponding author

E-mail: sabine.baumgartner@maastrichtuniversity.nl

**S3 Table. Fasting concentrations, iAUCs and maximal increases from baseline in apoB48 concentrations after consumption of a mixed meal containing no, or 3.0 gram of plant sterols or plant stanols separated per age category.**

|  | Control period | | | Sterol period | | | Stanol period | | |
| --- | --- | --- | --- | --- | --- | --- | --- | --- | --- |
|  | 18-35 y | 36-52 y | 53-69 y | 18-35 y | 36-52 y | 53-69 y | 18-35 y | 36-52 y | 53-69 y |
| Fasting (mg/L) | 12.9 ± 10.5 | 14.3 ± 9.2 | 11.2 ± 6.1 | 11.2 ± 7.6 | 12.9 ± 6.5 | 10.8 ± 4.6 | 11.1 ± 9.8 | 13.1 ± 6.9 | 10.8 ± 4.2 |
| iAUC^T^ (mg/L/min) | 2716.5 ± 1643 | 3360.3 ± 1664 | 3506.1 ± 2108 | 2754.7 ± 1874 | 2551.3 ± 1387 | 3833.9 ± 2495 | 2508.5 ± 1222 | 3262.9 ± 1637 | 4311.3 ± 1927 |
| iAUC^1^ (mg/L/min) | 1050.7 ± 470 | 1210.0 ± 547 | 1063.4 ± 651 | 1065.6 ± 507 | 967.5 ± 566 | 1124.5 ± 668 | 895.4 ± 445 | 1081.9 ± 717 | 1293.0 ± 600 |
| iAUC^2^ (mg/L/min) | 910.8 ± 636 | 1148.0 ± 747 | 1569.5 ± 978 | 1059.1 ± 706 | 796.9 ± 805 | 1328.7 ± 1151 | 1050.9 ± 789 | 1188.1 ± 626 | 2000.0 ± 1030^a,b^ |
| maxApoB48 (mg/L) | 11.3 ± 6.4 | 13.8 ± 5.2 | 15.1 ± 7.7 | 12.3 ± 7.1 | 10.8 ± 5.8 | 16.2 ± 9.4 | 11.1 ± 5.2 | 14.3 ± 7.8 | 18.0 ± 6.7^c^ |

Data are means ± SD

^a^ Significant difference stanol period compared with sterol period (*P* < 0.05); ^b^ trend for difference stanol period compared with control (*P* = 0.08); ^c^ significant difference stanol period compared with control and sterol period (P<0.05). Fasting concentrations in age categories II and III and iAUC^T^ and maxApoB48 in age category III were tested by Friedman’s test for not normally distributed data. iAUC^T^: incremental AUC of the total apoB48 response, iAUC^1^: incremental AUC after the 1^st^ meal (0-4h), iAUC^2^: incremental AUC after the 2^nd^ meal (4-8h)
