# Supplementary material for: Effects of a Plant Sterol or Stanol Enriched Mixed Meal on Postprandial Lipid Metabolism in Healthy Subjects
Source: PLoS One. 2016 Sep 9;11(9):e0160396. doi: 10.1371/journal.pone.0160396 (PMC5017646; doi:10.1371/journal.pone.0160396)
Supplement: S4 Table — (DOCX) [file pone.0160396.s007.docx]

Effects of a plant sterol or stanol enriched mixed meal on postprandial lipid metabolism in healthy subjects

Sabine Baumgartner^1^*, Ronald P. Mensink^1^ and Jogchum Plat^1^

^1^ Department of Human Biology, NUTRIM School of Nutrition and Translational Research in Metabolism, Maastricht University Medical Center, Maastricht, the Netherlands

* Corresponding author

E-mail: sabine.baumgartner@maastrichtuniversity.nl

**S4 Table. Fasting concentrations and AUCs of ApoCII, ApoCIII and ApoCIII/II values after consumption of a mixed meal containing no, or 3.0 gram of plant sterols or plant stanols separated per age category.**

|  | Control period | | | Sterol period | | | Stanol period | | |
| --- | --- | --- | --- | --- | --- | --- | --- | --- | --- |
|  | 18-35 y | 36-52 y | 53-69 y | 18-35 y | 36-52 y | 53-69 y | 18-35 y | 36-52 y | 53-69 y |
| ApoCII fasting (mg/dL) | 2.8 (1.5-5.8) | 4.3 (1.5-6.1) | 1.9 (2.2-7.7) | 2.6 (0.9-5.8) | 3.6 (1.3-5.8) | 4.1 (2.3-7.2) | 3.0 (1.2-5.4) | 3.3 (1.8-7.0) | 4.2 (1.5-6.7) |
| ApoCII AUC^T^ (mg/dL/min)^a^ | 1246 (670-2684) | 2074 (643-2993) | 1976 (924-3354) | 1286 (471-2767) | 1730 (542-2798) | 1871 (971-3260) | 1458 (593-2550) | 1583 (787-3367) | 1914 (672-3359) |
| ApoCII AUC^1^ (mg/dL/min)^a^ | 640 (384-1382) | 1039 (345-1523) | 1023 (497-1714) | 660 (245-1400) | 840 (307-1391) | 971 (519-1660) | 743 (290-1304) | 800 (440-1661) | 986 (358-1714) |
| ApoCII dAUC^2^ (mg/dL/min)^a^ | 40.0 (0.0-116.3) | 36.7 (5.2-57.2) | 24.6 (0.0-69.7) | 24.4 (0.0-110.6) | 21.7 (0.0-98.1) | 31.6 (0.8-142.0) | 34.8 (0.0-315.7) | 50.0 (0.0-114.7) | 53.9 (0.0-117.7) |
| ApoCIII fasting (mg/dL) | 9.1 (5.4-16.2) | 11.2 (5.8-17.1) | 10.4 (6.9-13.7) | 9.5 (4.1-15.3) | 9.2 (5.4-16.5) | 10.6 (7.2-15.7) | 8.6 (4.8-16.1) | 8.8 (6.3-13.7) | 10.3 (5.9-13.5) |
| ApoCIII AUC^T^ (mg/dL/min)^a^ | 4725 (2546-7708) | 4974 (2871-8329) | 4751 (2999-6386) | 4372 (1901-7521) | 4239 (2553-7772) | 5125 (2941-7361) | 4567 (2292-7590) | 3959 (3064-7125) | 4702 (2695-7014) |
| ApoCIII AUC^1^ (mg/dL/min)^a^ | 2270 (1263-3888) | 2562 (1437-4087) | 2346 (1537-3173) | 2206 (943-3652) | 2164 (1303-3863) | 2499 (1519-3614) | 2360 (1109-3804) | 1998 (1558-3397) | 2355 (1372-3353) |
| ApoCIII iAUC^2^ (mg/dL/min) | 34.9 (0.0-1610) | 33.3 (0.0-235.9) | 121.1 (0.0-224.8) | 68.4 (0.0-422.9) | 11.0 (0.0-308.3) | 90.5 (0.0-236.0) | 67.6 (0.0-325.8) | 119.9 (0.0-193.6) | 119.9 (15.0-353.1) |
| ApoCIII/II ratio fasting | 3.7 (2.6-5.3) | 2.7 (1.9-4.6) | 2.4 (1.6-4.4) | 3.4 (2.4-7.0) | 2.8 (2.1-4.2) | 2.7 (2.0-3.9) | 3.2 (2.4-5.5) | 2.6 (1.7-3.9) | 2.5 (1.9-4.0) |
| ApoCIII/II iAUC^T^ | 35.4 (0.0-283.6) | 15.8 (0.0-248.1) | 23.8 (2.2-65.0) | 47.4 (0.0-198.0) | 23.7 (0.0-328.6) | 35.0 (0.0-208.5) | 38.3 (1.8-270.2) | 72.8 (0.0-322.0) | 40.5 (10.9-238.0) |
| ApoCIII/II iAUC^1^ | 0.1 (0.0-42.0) | 0.0 (0.0-195.0) | 0.0 (0.0-16.8) | 8.1 (0.0-109.9) | 0.3 (0.0-49.6) | 0.3 (0.0-17.4) | 1.8 (0.0-112.3) | 2.7 (0.0-52.3) | 0.0 (0.0-3.3) |
| ApoCIII/II iAUC^2^ | 61.2 (5.5-259.8) | 41.0 (12.6-170.5) | 36.1 (6.3-114.0) | 40.0 (0.0-263.4) | 45.4 (0.0-124.2) | 52.9 (4.7-125.5) | 56.9 (3.8-375.3_ | 81.5 (0.0-160.4) | 72.2 (25.7-263.3)^b^ |

All parameters were tested by Friedman’s test for not normally distributed data and are presented as medians (ranges).

^a^ Responses are given in AUC or dAUC since time curves fluctuate below and above baseline levels. ^b^ Significant difference stanol period compared with control period (*P* < 0.05).

AUC^T^: AUC of the total apoC response, AUC^1^: AUC after the 1^st^ meal (0-4h), iAUC^2^: incremental AUC after the 2^nd^ meal (4-8h), dAUC^2^: decremental AUC after the 2^nd^ meal.
